# Identification of functional genes and immune cell infiltration analysis in liposarcoma

**DOI:** 10.1371/journal.pone.0358180

**Published:** 2026-09-11

**Authors:** Chunxiao Liu, Yanhua Wang, Qiuxia Liu, Sha Zhang

**Affiliations:** 1 Department of Cardiovascular Surgery, Qilu Hospital of Shandong University, Jinan, Shandong, China; 2 Department of Critical Care Unit, Shandong Provincial Hospital Affiliated to Shandong First Medical University, Jinan, Shandong, China; Huazhong Agriculture University, CHINA

## Abstract

Liposarcoma (LPS) is a common soft tissue sarcoma; however, its molecular pathogenesis and immune cell infiltration remain poorly understood. This study investigated potential driver genes and pathways in LPS and characterized immune cell infiltration patterns to identify potential markers for targeted therapy. Differentially expressed genes (DEGs) in LPS were analyzed by GO and KEGG pathway enrichment analysis. A protein-protein interaction network was constructed using the STRING database and visualized with Cytoscape. mRNA expression of genes with high |logFC| values was verified by RT-qPCR. Immune cell subsets were quantified using CIBERSORT. GO and KEGG analysis revealed significant functional clusters and pathways, and most verified genes were consistent with the bioinformatics analysis. Survival analysis showed that high expression of TYMS, KIF20A, BUB1B, LMNB1, RRM2, ZWINT, and RACGAP1 was significantly associated with poor overall survival and poor disease-free survival (DFS). High levels of TMSB15A, TPX2, PKM2, and PTTG1 were significantly associated with poor DFS alone. CIBERSORT analysis identified a significantly higher fraction of resting mast cells (MCs) in LPS tissues compared to normal fatty tissues (P < 0.05). TOP2A, IL-6, PCNA, CDK1, JUN, MYC, CCNB1, EGFR, ACACB, and BIRC5 were identified as potential diagnostic biomarkers of LPS, providing strong evidence for hub gene studies. Immune cell infiltration analysis further suggested that resting MCs may play a potential role in LPS development, although further experimental validation is warranted. Collectively, these findings clarify the molecular basis of LPS and provide a foundation for future research into its treatment.

## Introduction

Liposarcoma (LPS) is one of the most common soft tissue sarcomas, which accounts for ~20% of all soft tissue sarcomas [1]. The 2020 WHO Classification of Soft Tissue Tumors recognizes five histological subtypes: Well-differentiated (WDLPS), de-differentiated (DDLPS), myxoid (MLPS), pleomorphic (PLPS) and myxoid pleomorphic LPS (MPLPS) [2]. WDLPS and DDLPS collectively account for the majority of LPS cases, which are featured by chromosome amplification at 12q13-q22 that contains MDM2 and CDK4 genes [3]. DDLPS can evolve from the progression of primary WDLPS and acquire greater recurrence and metastasis potential [4]. MLPS accounts for 5–10% of soft tissue sarcomas and, in patients aged <20 years, it is the most frequent LPS subtype [5]. PLPS is the rarest subtype that commonly arises in the pelvis and thigh of old patients [6]. Despite being the only curative option, surgical resection is often not possible for advanced LPS. Furthermore, treatment is hampered by subtype heterogeneity, which dictates diverse biological behaviors and variable responses to radiotherapy and chemotherapy [7]. It is therefore urgent to identify the molecular mechanisms underlying the development and progression of LPS and to identify promising therapeutic targets.

Microarray chips have facilitated the generation of high-throughput data, advancing genetic research. Bioinformatics analysis of microarray data comparing gene expression and functional pathways between patients and healthy donors offers a powerful strategy to understand pathogenesis and identify novel therapeutic targets. In the present study, the potential driver genes and pathways of LPS were analyzed using bioinformatics analysis, building upon our previous study of the public dataset GSE21122, in which DEGs associated with LPS were identified [8], and immune cell infiltration analysis was performed for future research and treatment.

## Materials and methods

### Data processing and differential gene identification

The differentially expressed genes (DEGs) analyzed in the present study were derived from a previously published dataset (GSE21122) and our earlier analysis [8]. Briefly, the raw microarray data were processed using the RMA normalization algorithm, and differential expression was evaluated using the limma package with an empirical Bayes approach. Genes with an FDR < 0.05 and a |log2FC| > 1 were considered as DEGs.

### Gene ontology (GO) and pathway enrichment analysis

The DAVID database and clusterprofiler R package were used to perform GO and Kyoto Encyclopedia of Genes and Genomes (KEGG) pathway analysis. FDR < 0.05 was used as the cut-off criterion.

### Protein-protein interaction (PPI) network and hub gene selection

PPI networks on DEGs were constructed and assessed using the Search Tool for theRetrieval of Interacting Genes (STRING) database, and visualized using Cytoscape software (Version 3.10.2). Molecular complex detection was performed to screen modules of the PPI network with MCODE of Cytoscape software. Hub genes were identified as the top 10 genes ranked by node degree in the PPI network. Node degree reflects the number of direct interactions a gene has with other genes in the network. The feature selection of hub genes was performed using the cytoHubba plugin in Cytoscape.

### Reverse transcription-quantitative PCR (RT-qPCR)

A total of 13 retroperitoneal LPS (RPLPS) samples and 10 normal fatty (NF) tissues were collected from patients who underwent surgical resection at Shandong Provincial Hospital (Shandong, China) between March 2021 and February 2022. Patient characteristics were summarized in S1 Table. The present study was approved by the Ethics Committee of Shandong Provincial Hospital (Approval No. SZRJJ:NO.2021−037), and written informed consent was obtained from each participant. The study was conducted in accordance with the Declaration of Helsinki. The research activities, including RNA extraction, RT-qPCR analysis, and data analysis, were conducted between February 2022 and March 2022. Patient samples were coded and anonymized before analysis. The authors did not have access to identifying information during or after data analysis. None of the patients had received radiotherapy or chemotherapy prior to surgery, and all tumor samples were pathologically confirmed as RLPS. Candidate genes for RT-qPCR validation were selected from the DEGs based on two criteria: high |log_2_FC| values (ranking among the top upregulated or downregulated genes) and documented biological significance in cancer or liposarcoma based on previous literature. Total RNA was extracted using TRIzol^®^ reagent (cat no.15596026, Invitrogen, Thermo Fisher Scientific, Inc.), and cDNA was synthesized using a reverse transcription kit (5X All-In-One RT MasterMix Kit, cat. no. G490, ABM), according to the manufacturer’s instructions. Primers were designed using Primer 6.0 (Applied Biosystems Inc.). Genes and primer sequences are shown in S2 Table. The mRNA expression of candidate genes was measured using the Applied Biosystems 7500 Fast Real-Time PCR System (Applied Biosystems, Thermo Fisher Scientific, Inc.) with SYBR® Green Real-time PCR Master Mix kit (ABM, EvaGreen 2X qPCR MasterMix-Low ROX, MasterMix-LR), according to the manufacturer’s instructions. GAPDH was used as an internal reference gene. All reactions were performed in triplicate. The specificity of RT-qPCR was confirmed by melting-curve analysis. The relative expression is calculated and analyzed using the value of each gene and GAPDH according to the 2^-ΔΔCt^ formula.

### Survival analysis

Survival analysis and curves were evaluated using the Gene Expression Profiling Interactive Analysis (GEPIA) online platform (http://gepia.cancer-pku.cn/?from=timeline&isappinstalled=0), which is based on RNA-seq data from The Cancer Genome Atlas (TCGA) and genotype-tissue expression (GTEx) projects. Differential expression analysis between sarcoma tumors (n = 263) and normal tissues (n = 737) was performed with parameters set as |Log2FC| > 1 and P < 0.01. For survival analysis, patients were automatically stratified into ‘high-expression’ and ‘low-expression’ groups based on the median expression value of each gene within the SARC cohort. Overall survival (OS) and disease-free survival (DFS) were used as the survival endpoints. Statistical significance was assessed using the Log-rank test. Due to the limited sample size of individual liposarcoma subtypes within the TCGA-SARC cohort, subtype‑specific survival analysis was not feasible; therefore, the analysis was performed on the combined sarcoma cohort. This limitation is acknowledged in the Discussion section.

### Evaluation and correlation analysis of infiltrating immune cells

For each sample, CIBERSORT analysis was performed using R package “e1071” with the default LM22 gene signature matrix and 1,000 permutations to determine the relative abundance of immune cell populations. Only samples with CIBERSORT P < 0.05 were retained for subsequent analysis. No multiple comparison correction was applied to the immune cell fractions, as the analysis was exploratory in nature. The link between hub gene expression and immune cell infiltration levels was evaluated using the R package “tidyverse”.

### Statistical analysis

Statistical analysis was performed using SPSS version 27 (IBM Corp.) and GraphPad Prism version 10 (GraphPad Software, Inc.). An independent samples t‑test was performed to compare two groups of quantitative data. P < 0.05 was considered to indicate a statistically significant difference.

## Results

### Identification of DEGs in LPS

**Fig 1:**
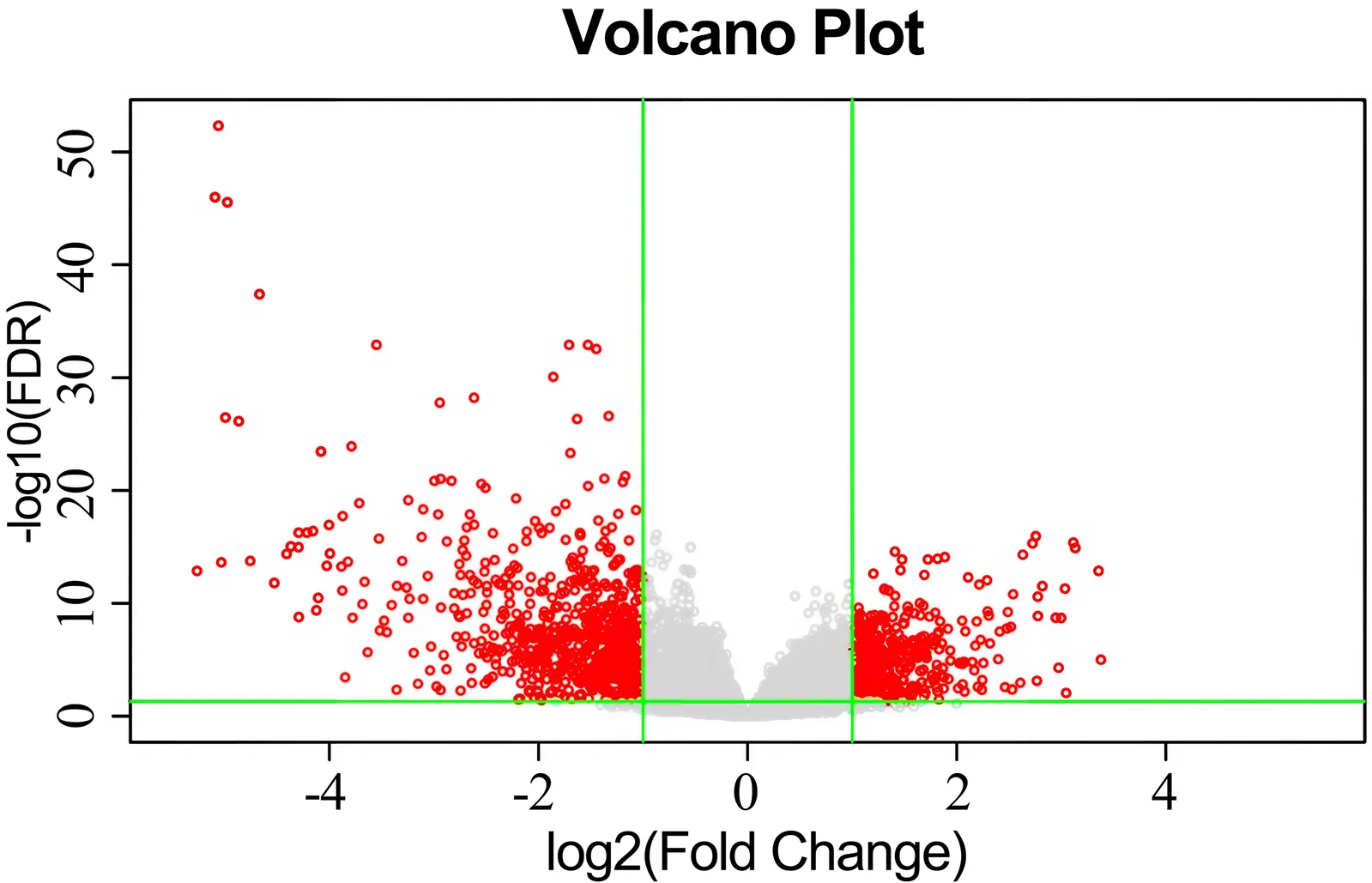
The differentially expressed genes that were identified between LPS and NF tissues. A total of 855 differentially expressed genes (DEGs) were extracted from the expression profile dataset of GSE21122, of which 334 were upregulated genes and 521 were downregulated genes.

The microarray data of GSE21122 contains 89 LPS tissues (including 46 DDLPS, 20 MLPS and 23 PLPS) and 9 NF tissues. FDR < 0.05 and |logFC| > 1 was defined as the cut-off criterion. A total of 855 DEGs were extracted from the expression profile dataset of GSE21122, wherein 334 genes were upregulated and 521 downregulated [8]. The collagen type I alpha 1 chain (COL1A1) gene was an upregulated gene with the highest |log FC|, and CDC28 protein kinase regulatory subunit 2 (CKS2), thymidylate synthetase (TYMS), proliferating cell nuclear antigen (PCNA), clamp associated factor, delta like non-canonical Notch ligand 1 (DLK1), neuronal regeneration related protein, Zic family zinc finger 1 (ZIC1), serpin family E member 2, ribonucleotide reductase regulatory subunit M2 (RRM2) and COL5A1 genes were also upregulated, with the their |log FC| being at the top of the list. The perilipin 1 (PLIN1) gene was a downregulated gene with the highest |log FC|, and serum amyloid A (SAA)2-SAA4, solute carrier family 19 member 3 (SLC19A3), adipogenesis regulatory factor, protein phosphatase 1 regulatory inhibitor subunit 1A (PPP1R1A), SAA1, SAA2, cell death inducing DFFA like effector c (CIDEC), hemoglobin subunit beta and solute carrier family 19 member 3 (CIDEA) were downregulated genes with the |log FC| being at the top of the list (Table 1). A volcano plot shows the differential distribution of these 855 DEGs (Fig 1).

**Table 1:** The significant DEGs with the |log FC| at the top of the list.

Gene symbol	logFC	|log FC|	P.Value	FDR
COL1A1	3.378763	3.378763	6.20E-07	9.93E-06
CKS2	3.356810	3.356810	9.40E-16	1.34E-13
TYMS	3.134542	3.134542	5.36E-18	1.27E-15
KIAA0101	3.115156	3.115156	1.61E-18	4.17E-16
DLK1	3.046623	3.046623	1.97E-03	1.97E-03
NREP	3.035670	3.035670	5.39E-14	4.96E-12
ZIC1	2.998437	2.998437	3.82E-11	1.98E-09
SERPINE2	2.974046	2.974046	4.13E-06	5.10E-05
RRM2	2.819026	2.819026	3.05E-14	2.98E-12
COL5A1	2.776362	2.776362	2.47E-11	1.35E-09
PLIN1	−5.266206	5.266206	9.52E-16	1.34E-13
SAA2-SAA4	−5.094886	5.094886	1.20E-50	1.04E-46
SLC19A3	−5.061968	5.061968	2.80E-57	4.87E-53
ADIRF	−5.034183	5.034183	1.36E-16	2.37E-14
PPP1R1A	−4.995028	4.995028	2.57E-30	3.43E-27
SAA1	−4.975437	4.975437	5.09E-50	2.95E-46
SAA2	−4.975437	4.975437	5.09E-50	2.95E-46
CIDEC	−4.865646	4.865646	6.33E-30	7.33E-27
HBB	−4.757226	4.757226	9.64E-17	1.76E-14
CIDEA	−4.668126	4.668126	9.48E-42	4.12E-38

### GO enrichment analysis in LPS

**Fig 2:**
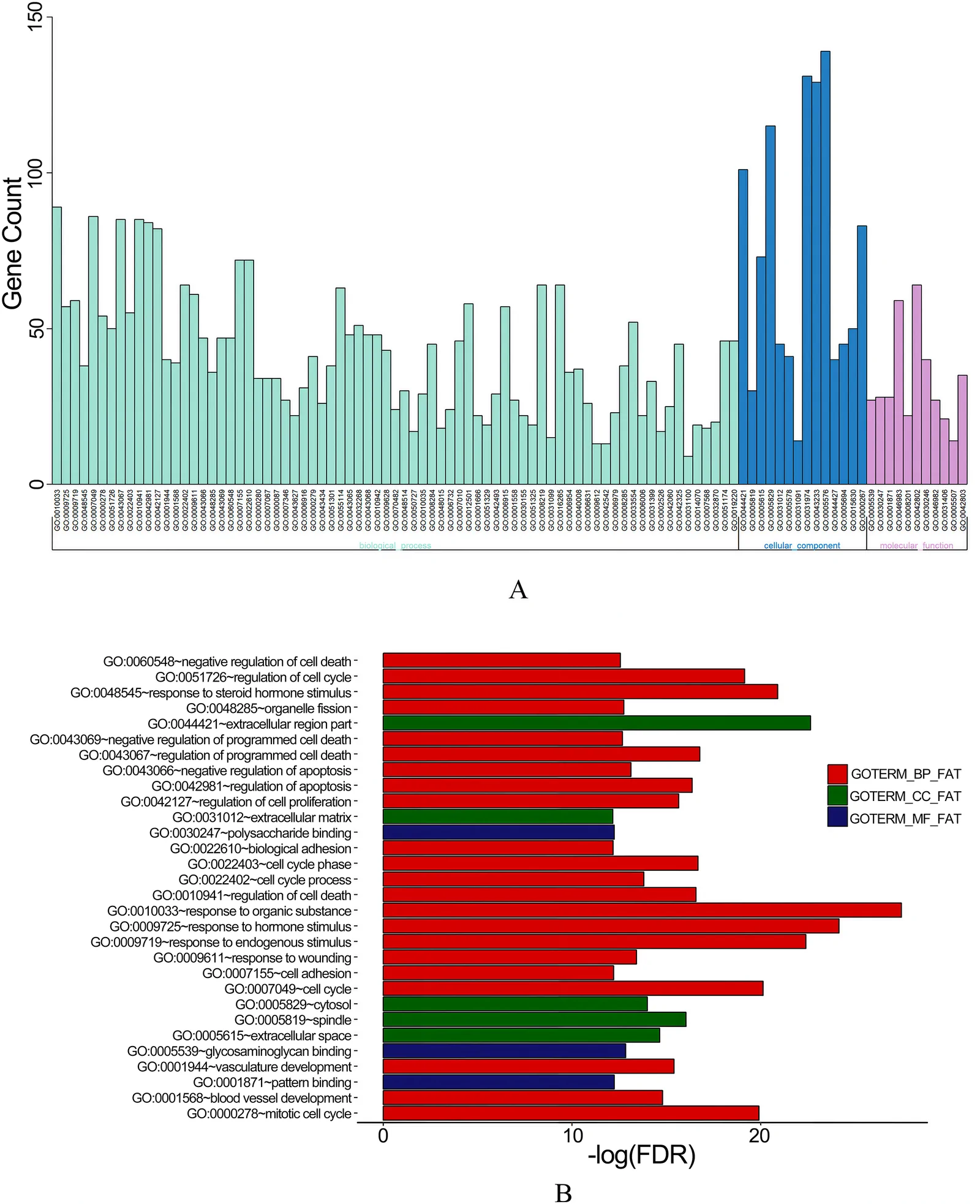
Gene Ontology analysis and significantly enriched GO terms of DEGs in LPS. (A) Three functional groups of GO function enrichment analysis: biological process group (BP), cellular component group (CC), and molecular function group (MF). (B) Significant Go Terms of DEGs in LPS based on their functions. The functional group of biological process accounted for the vast majority of the top 30 GO terms according to FDR.

The GO function enrichment includes three functional groups: Molecular function group (MF), biological process group (BP) and cellular component group (CC) (Fig 2A). As shown in Fig 2B, the functional group of BP accounted for the vast majority of the top 30 GO terms according to FDR. The top 10 GO terms in which upregulated genes were highly enriched were as follows: Non-membrane-bounded organelle (CC), intracellular non-membrane-bounded organelle (CC), organelle lumen (CC), membrane-enclosed lumen (CC), intracellular organelle lumen (CC), cell cycle (BP), nuclear lumen (CC), cytoskeleton (CC), cell cycle process (BP) and mitotic cell cycle (BP). The top 10 GO terms in which downregulated genes were highly enriched as follows: Plasma membrane part (CC), extracellular region (CC), cytosol (CC), response to organic substance (BP), extracellular region part (CC), cell fraction (CC), regulation of programmed cell death (BP), regulation of cell death (BP), regulation of apoptosis (BP) and regulation of cell proliferation (BP) (Table 2). These results indicated that most of the DEGs were significantly enriched in organelle component, membrane component and cell cycle functional clusters.

**Table 2:** The top 10 most enriched GO terms of DEGs in LPS.

Term	Description	Count	FDR
**Up-regulated**			
GO:0043228	non-membrane-bounded organelle	95	1.43E-09
GO:0043232	intracellular non-membrane-bounded organelle	95	1.43E-09
GO:0043233	organelle lumen	71	3.37E-07
GO:0031974	membrane-enclosed lumen	71	8.08E-07
GO:0070013	intracellular organelle lumen	69	8.92E-07
GO:0007049	cell cycle	65	6.73E-23
GO:0031981	nuclear lumen	61	6.15E-07
GO:0005856	cytoskeleton	53	2.78E-04
GO:0022402	cell cycle process	52	3.22E-19
GO:0000278	mitotic cell cycle	46	6.79E-22
**Down-regulated**			
GO:0044459	plasma membrane part	100	0.023491
GO:0005576	extracellular region	92	0.043886
GO:0005829	cytosol	80	3.40E-06
GO:0010033	response to organic substance	71	6.95E-15
GO:0044421	extracellular region part	67	2.54E-07
GO:0000267	cell fraction	62	0.001913
GO:0043067	regulation of programmed cell death	60	1.07E-06
GO:0010941	regulation of cell death	60	1.23E-06
GO:0042981	regulation of apoptosis	59	2.01E-06
GO:0042127	regulation of cell proliferation	58	2.46E-06
FDR = false discovery rate, GO = Gene Ontology.			

### Signaling pathway enrichment analysis in LPS

**Fig 3:**
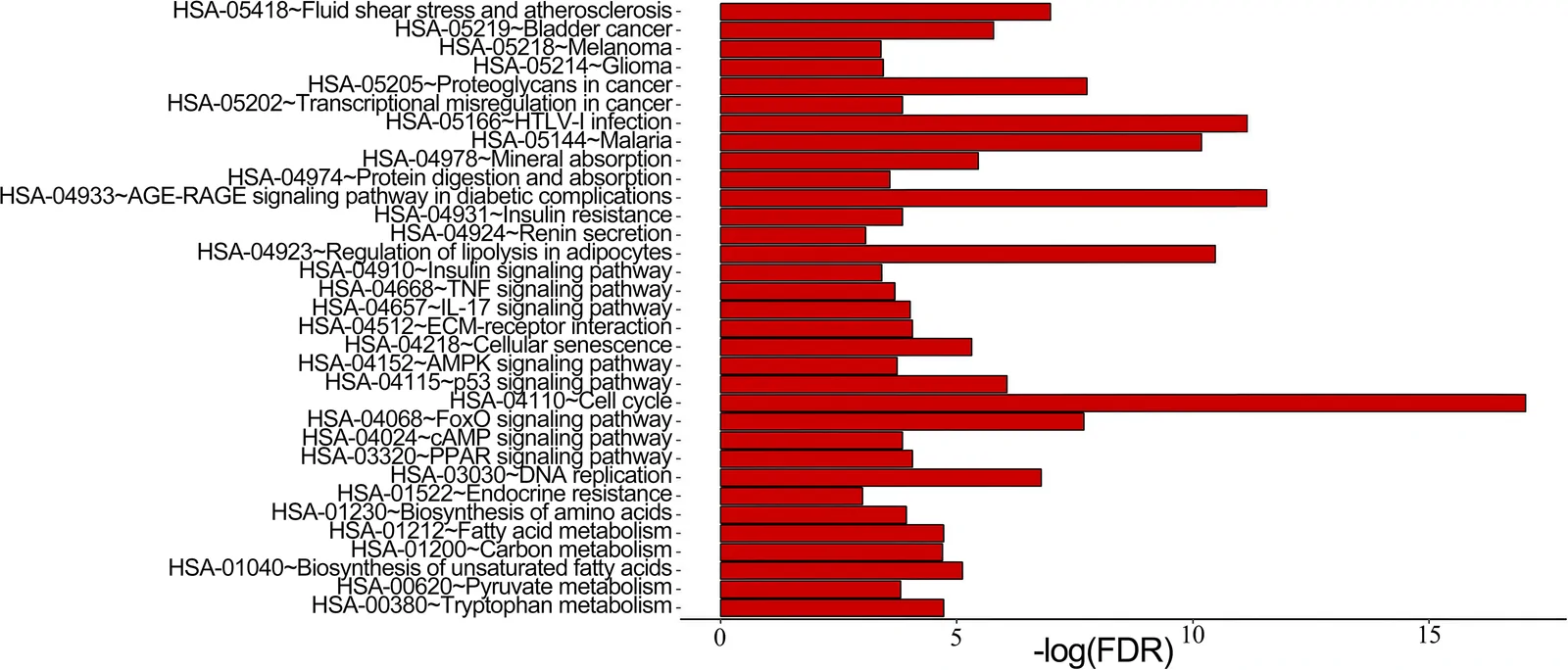
Significantly enriched pathway terms of DEGs in LPS. The 5 most significant pathways were cell cycle, AGE-RAGE signaling pathway in diabetic complications, HTLV-I infection, regulation of lipolysis in adipocytes and malaria.

The screened DEGs were used to perform signaling pathway enrichment analysis, and the five most significant pathways were cell cycle, AGE-RAGE signaling pathway in diabetic complications, HTLV-I infection, regulation of lipolysis in adipocytes and malaria (Fig 3). The upregulated genes were highly enriched in cell cycle, HTLV-I infection, human papillomavirus infection, DNA replication and oocyte meiosis pathways. The downregulated genes majorly enriched in fluid shear stress and atherosclerosis, HTLV-I infection, cAMP signaling pathway, proteoglycans in cancer, and kaposi sarcoma-associated herpesvirus infection pathways (Table 3). The common pathway of up- and downregulated genes was enriched in HTLV-I infection, which could induce tumorigenesis by activating oncogenes in the host cell.

**Table 3:** Signaling pathway enrichment analysis of DGEs in LPS.

Term	Description	Count	FDR	Genes
**Upregulated**				
HSA-04110	Cell cycle	24	9.2981E-16	PTTG1, SKP2, CDK4, CDC20, MAD2L1, CDC7, PRKDC, ORC5, MCM2, SMC1A, CDKN2A, CCNB1, CCNB2, CDC23, MCM4, MCM5, CDC25B, CDK1, MCM6, PCNA, BUB1B, MDM2, CCNA2, TTK
HSA-05166	HTLV-I infection	18	0.000104041	PTTG1, CDK4, CDC20, FZD1, MAD2L1, BAX, XPO1, FZD7, CDKN2A, FZD2, CCNB2, WNT5A, CDC23, ADCY1, CALR, PCNA, BUB1B, POLE2
HSA-05165	Human papillomavirus infection	15	0.033882048	PKM, CDK4, FZD1, BAX, FZD7, COL6A2, FZD2, COL1A1, COL6A1, ISG15, WNT5A, COL1A2, MDM2, CCNA2, FN1
HSA-03030	DNA replication	10	8.04325E-08	FEN1, RFC4, RFC3, MCM2, LIG1, MCM4, MCM5, MCM6, PCNA, POLE2
HSA-04114	Oocyte meiosis	10	0.003486832	PTTG1, CDC20, MAD2L1, SMC1A, CCNB1, CCNB2, CDC23, ADCY1, CDK1, AURKA
**Downregulated**				
HSA-05418	Fluid shear stress and atherosclerosis	20	7.49066E-05	CDH5, CCL2, IL1R2, THBD, NQO1, CALM1, JUN, GSTT1, ASS1, CAV1, CALM2, MAP3K5, IL1R1, CAV2, DUSP1, FOS, BCL2, KLF2, PECAM1, ICAM1
HSA-05166	HTLV-I infection	20	0.03492525	RRAS2, ZFP36, RRAS, MYC, IL1R2, STAT5B, JUN, EGR1, MRAS, NFKBIA, CDKN1A, ATF3, IL6, IL1R1, FZD4, HLA-E, FOS, TLN2, ETS2, ICAM1
HSA-04024	cAMP signaling pathway	19	0.007970318	RRAS2, RRAS, ADRB2, CALM1, LIPE, JUN, MYL9, NPR1, FXYD1, NFKBIA, CALM2, ACOX1, RAPGEF3, PTGER3, GNAI1, PLN, HCAR3, FOS, PDE3B
HSA-05205	Proteoglycans in cancer	19	0.008547244	FGF2, EGFR, RRAS2, ANK2, RRAS, MET, MYC, TIMP3, HSPB2, MRAS, CAV1, CDKN1A, HBEGF, THBS1, FZD4, CAV2, PTPN11, CD44, IGF1
HSA-05167	Kaposi sarcoma-associated herpesvirus infection	17	0.017966457	FGF2, ZFP36, MYC, CALM1, IL6ST, JUN, NFKBIA, CALM2, CDKN1A, CXCL8, C3, GABARAPL1, IL6, CXCL2, HLA-E, FOS, ICAM1

### PPI and modular analysis

**Fig 4:**
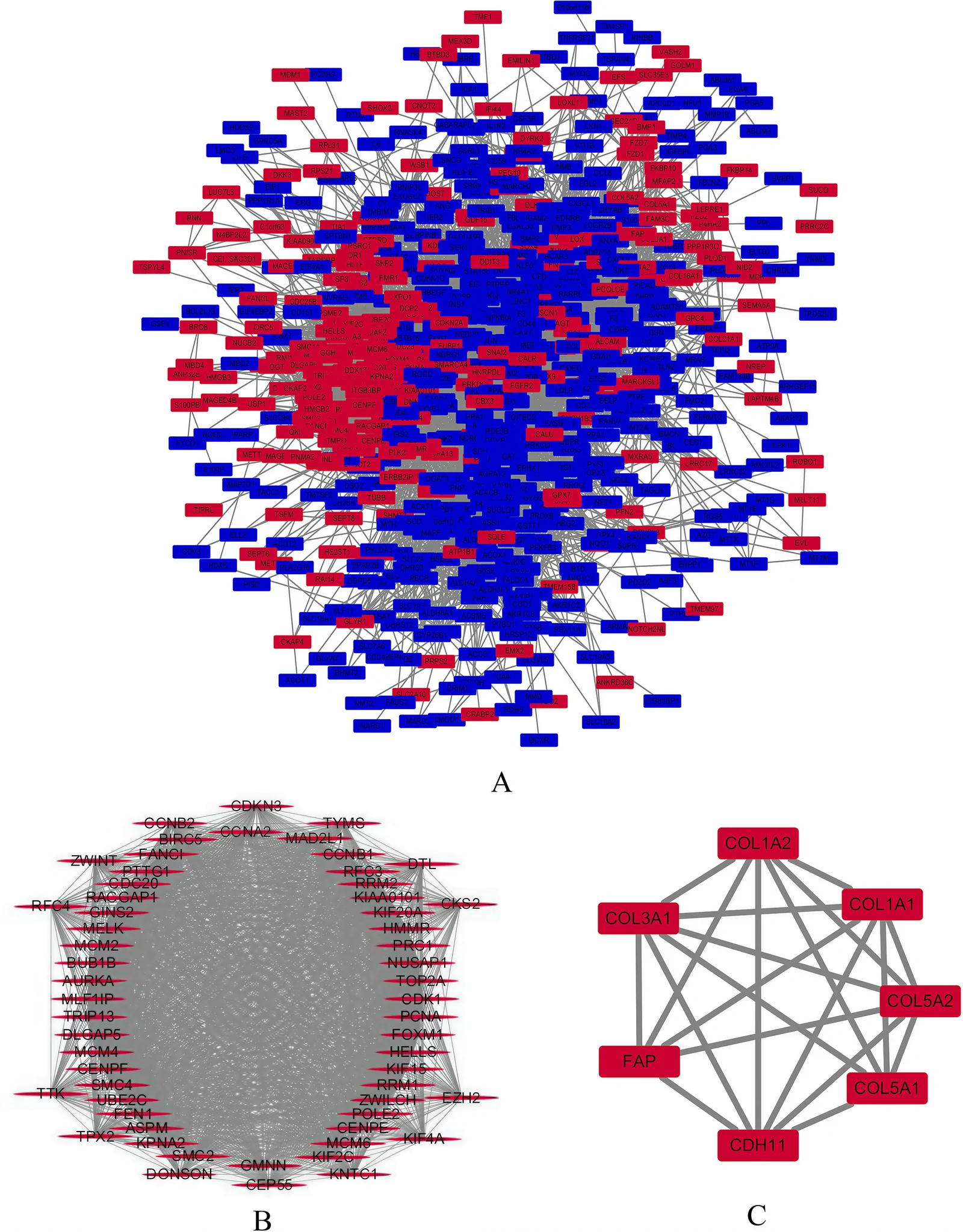
PPI network and module analysis. (A) PPI network complex. A total of 821 nodes with 7200 edges were mapped in the PPI network after the disconnected genes were excluded. The red points represent upregulated genes, and the blue points represent downregulated genes. Two important modules were selected from PPI network complex (B) Module 1 was composed of 58 nodes and 1578 edges. (C) Module 2 was composed of 7 nodes and 20 edges.

All 855 DEGs were used to construct PPI network. A total of 821 nodes with 7,200 edges were mapped in the PPI network after the disconnected genes were excluded (Fig 4A). The top 10 node-degree genes were DNA topoisomerase II alpha (TOP2A), IL-6, PCNA cyclin dependent kinase 1 (CDK1), Jun proto-oncogene (JUN), v-myc avian myelocytomatosis viral oncogene homolog (MYC), cyclin B1 (CCNB1), epidermal growth factor receptor (EGFR), acetyl-CoA carboxylase beta (ACACB), baculoviral IAP repeat containing 5 (BIRC5). The top 10 hub genes identified from the PPI network were considered as potential diagnostic biomarkers for liposarcoma. Two important modules were selected from PPI network complex and Cytoscape MCODE was used for further analysis. Module 1, containing 58 nodes and 1,578 edges (Fig 4B), was significantly enriched for terms associated with the cytoplasm and intracellular components (Table 4). By contrast, the smaller Module 2 (7 nodes, 20 edges; Fig 4C) was primarily associated with processes such as locomotion and cellular movement (Table 5).

**Table 4:** Pathway enrichment analysis of Module 1 gene function.

Term	Description	Count	FDR
GO.0005737	cytoplasm	47	6.49E-07
GO.0044424	intracellular part	47	0.00152
GO.0005622	intracellular	47	0.00339
GO.0007049	cell cycle	46	6.70E-42
GO.0000278	mitotic cell cycle	45	5.87E-49
GO.0044446	intracellular organelle part	45	7.99E-10
GO.0016043	cellular component organization	44	2.70E-16
GO.0043229	intracellular organelle	43	0.00371
GO.0022402	cell cycle process	42	8.56E-40
GO.0005634	nucleus	42	3.84E-09
GO.0006996	organelle organization	41	3.71E-21
GO.0044763	single-organism cellular process	40	0.00414
GO.1903047	mitotic cell cycle process	39	3.06E-40
GO.0031981	nuclear lumen	37	2.46E-14
GO.1902589	single-organism organelle organization	36	6.47E-21
GO.0032991	macromolecular complex	36	4.01E-10
GO.0005654	nucleoplasm	35	1.35E-14
GO.0043232	intracellular non-membrane-bounded organelle	35	3.32E-12
GO.0044428	nuclear part	35	9.93E-12
GO.0043234	protein complex	35	4.30E-11
GO.0050896	response to stimulus	34	0.000406
GO.0044444	cytoplasmic part	33	0.00384
GO.0051716	cellular response to stimulus	31	0.000529
GO.0051301	cell division	30	3.03E-31
GO.0044260	cellular macromolecule metabolic process	29	0.0342
GO.0003824	catalytic activity	28	0.00525
GO.1901363	heterocyclic compound binding	28	0.0144
GO.0097159	organic cyclic compound binding	28	0.0173
GO.0005829	cytosol	28	7.03E-08
GO.0007067	mitotic nuclear division	26	2.33E-28

**Table 5:** Pathway enrichment analysis of Module 2 gene function.

Term	Description	Count	FDR
GO.0040011	locomotion	6	0.000123
GO.0006928	movement of cell or subcellular component	6	0.000217
GO.0009056	catabolic process	6	0.000902
GO.0044421	extracellular region part	6	0.0177
GO.0005576	extracellular region	6	0.0413
GO.0030199	collagen fibril organization	5	4.86E-09
GO.0030574	collagen catabolic process	5	5.44E-08
GO.0022617	extracellular matrix disassembly	5	1.84E-07
GO.0043588	skin development	5	3.28E-06
GO.0001568	blood vessel development	5	8.43E-05
GO.0001944	vasculature development	5	9.94E-05
GO.0051239	regulation of multicellular organismal process	5	0.0448
GO.0005201	extracellular matrix structural constituent	5	1.10E-08
GO.0005583	fibrillar collagen trimer	5	4.56E-13
GO.0098643	banded collagen fibril	5	4.56E-13
GO.0005788	endoplasmic reticulum lumen	5	4.32E-07
GO.0005783	endoplasmic reticulum	5	4.76E-03
KEGG Pathway HAS-4512	ECM-receptor interaction	5	3.29E-09
KEGG Pathway HAS-4974	Protein digestion and absorption	5	3.29E-09
KEGG Pathway HAS-5146	Amoebiasis	5	6.14E-09
KEGG Pathway HAS-4611	Platelet activation	5	1.22E-08
KEGG Pathway HAS-4510	Focal adhesion	5	1.06E-07
KEGG Pathway HAS-4151	PI3K-Akt signaling pathway	5	1.13E-06
GO.0071230	cellular response to amino acid stimulus	4	1.60E-06
GO.0001501	skeletal system development	4	0.00294
GO.0007409	axonogenesis	4	0.00369
GO.0061564	axon development	4	0.00416
GO.0048667	cell morphogenesis involved in neuron differentiation	4	0.00459
GO.0042060	wound healing	4	0.00988
GO.0016477	cell migration	4	0.0133

### mRNA expression of representative DEGs

**Fig 5:**
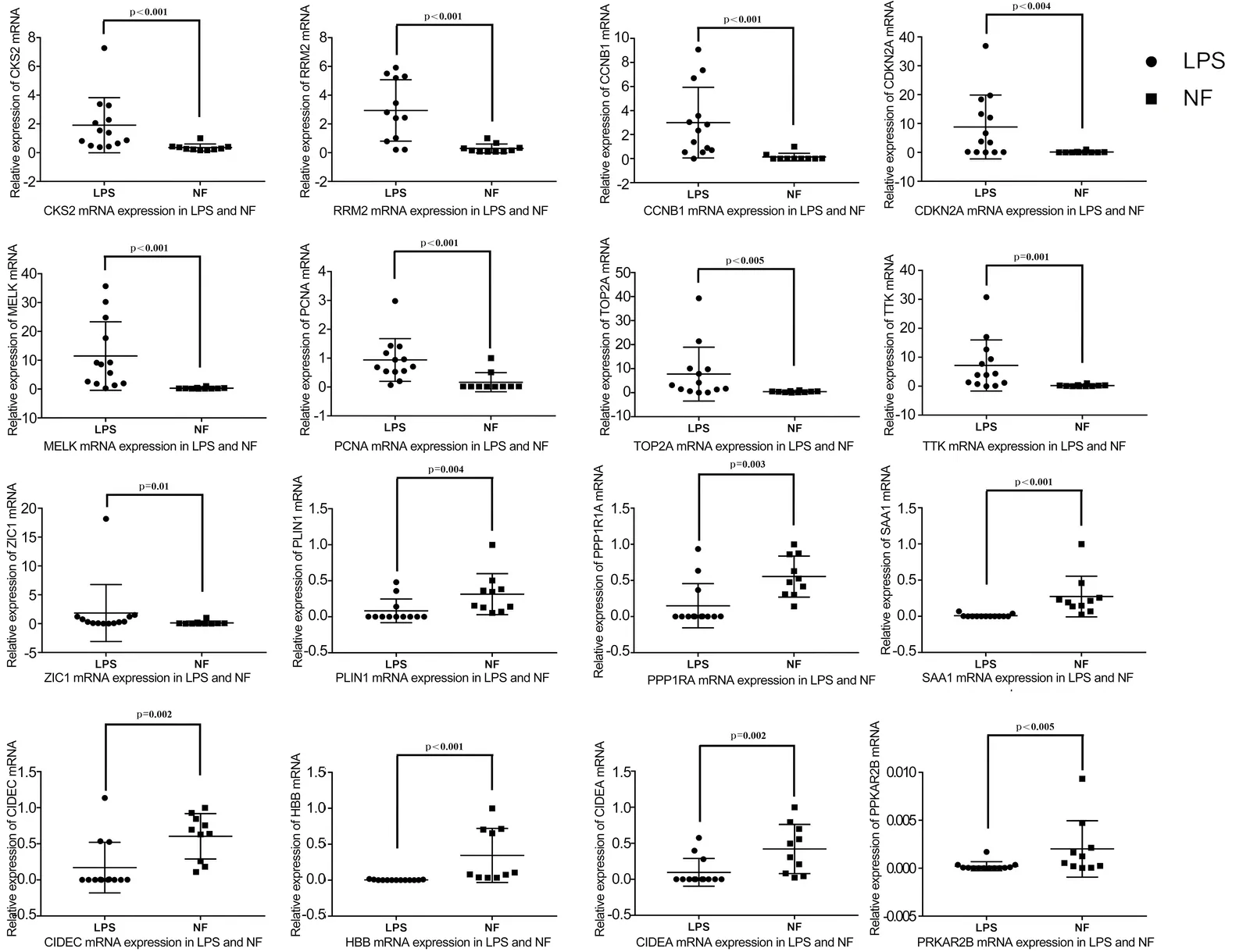
The mRNA expression level of some representative genes with high |logFC| values was verified in LPS and NF by using real-time PCR. A total of 13 retroperitoneal liposarcoma (RPLPS) tissues and 10 normal fatty (NF) tissues were collected for real-time PCR. The mRNA expression of CKS2, RRM2, CCNB1, CDKN2A, MELK, PCNA, TOP2A, TTK, ZIC1 was upregulated and the mRNA expression of PLIN1, PPP1R1A, SAA1, CIDEC, HBB, CIDEA, PRKAR2B was downregulated, which was consistent with the results of our bioinformatics analysis.

**Fig 6:**
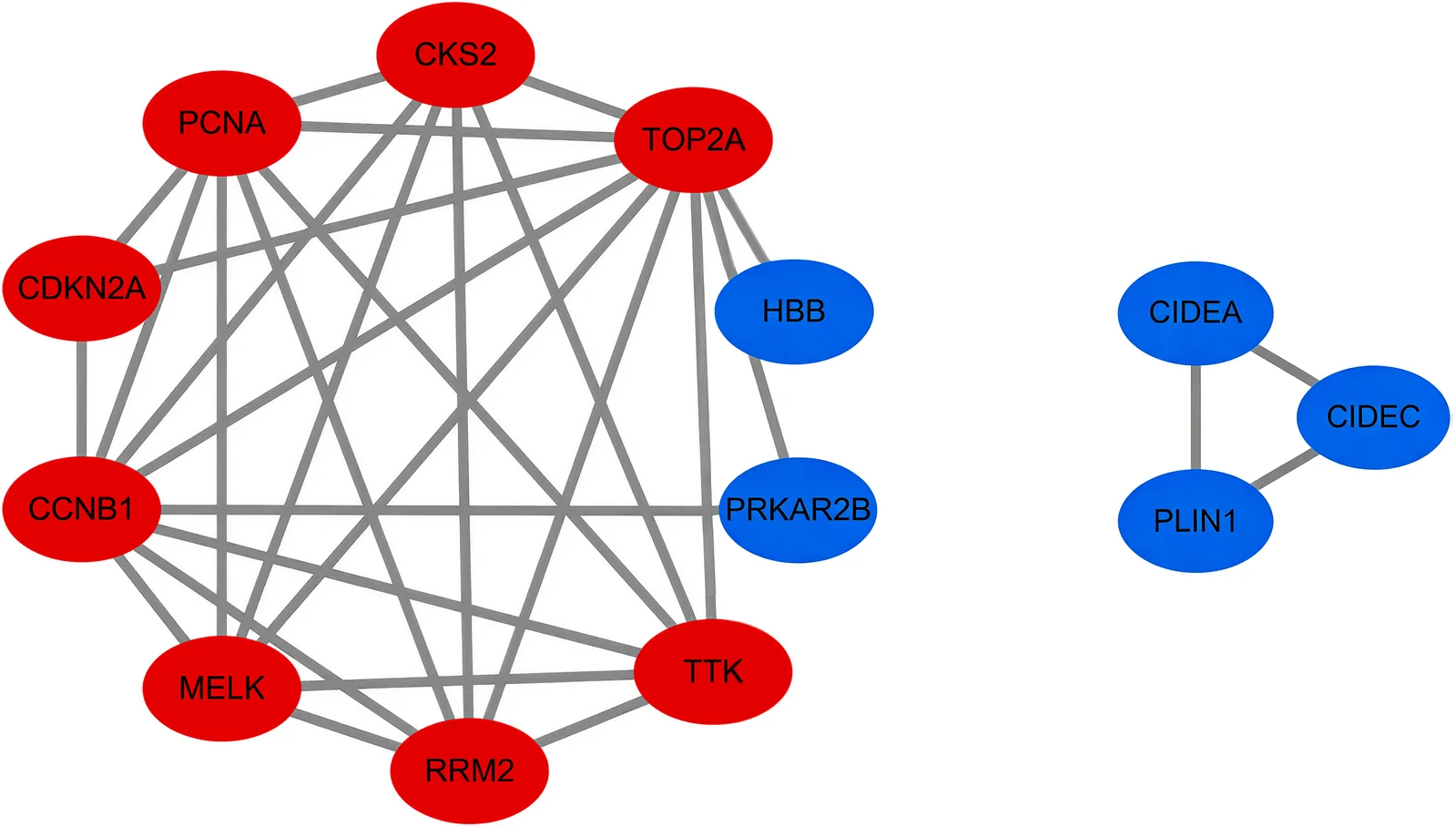
Small regulatory network formed by verified genes. Thirteen of the eighteen genes that the mRNA expression level was identified between LPS and NF tissues formed two small regulatory networks, the genes which were parts of the general network that was constructed by all DEGs.

A preliminary test on representative DEGs was performed using RT-qPCR to verify their differential expression in LPS and NF. The mRNA expression of representative upregulated genes [COL1A1, CKS2, ribonucleotide RRM2, CCNB1, cyclin dependent kinase inhibitor 2A (CDKN2A), maternal embryonic leucine zipper kinase (MELK), PCNA, TOP2A, TTK protein kinase (TTK) and ZIC1] and downregulated genes [PLIN1, SLC19A3, PPP1R1A, SAA1, CIDEC, HBB, CIDEA, protein kinase cAMP-dependent type II regulatory subunit beta (PRKAR2B)] was examined. It was found that CKS2, RRM2, CCNB1, CDKN2A, MELK, PCNA, TOP2A, TTK and ZIC1 (P < 0.001, P < 0.001, P < 0.001, P = 0.004, P < 0.001, P < 0.001, P = 0.005, P = 0.001 and P = 0.01 respectively) were upregulated and PLIN1, PPP1R1A, SAA1, CIDEC, HBB, CIDEA and PRKAR2B (P = 0.004, P = 0.003, P < 0.001, P = 0.002, P < 0.001, P = 0.002 and P = 0.005, respectively) were downregulated in LPS compared with NF (Fig 5), which was consistent with the results of the bioinformatics analysis. There was no statistical difference for the expression of COL1A1 and SLC19A3 between LPS and NF (P > 0.05). The CKS2, RRM2, CCNB1, CDKN2A, MELK, PCNA, TOP2A, TTK, PLIN1, CIDEC, HBB, CIDEA and PRKAR2B genes formed two small regulatory networks (Fig 6), which were parts of the general network, and the GO terms of these 13 genes were enriched in protein kinase binding, cytoplasm and apoptotic process in MF, CC and BP, respectively. In addition, the pathways of these 13 genes were mainly involved in cell cycle and p53 signaling pathway.

### Correlation between prognosis and proliferation-related genes in sarcoma patients

**Fig 7:**
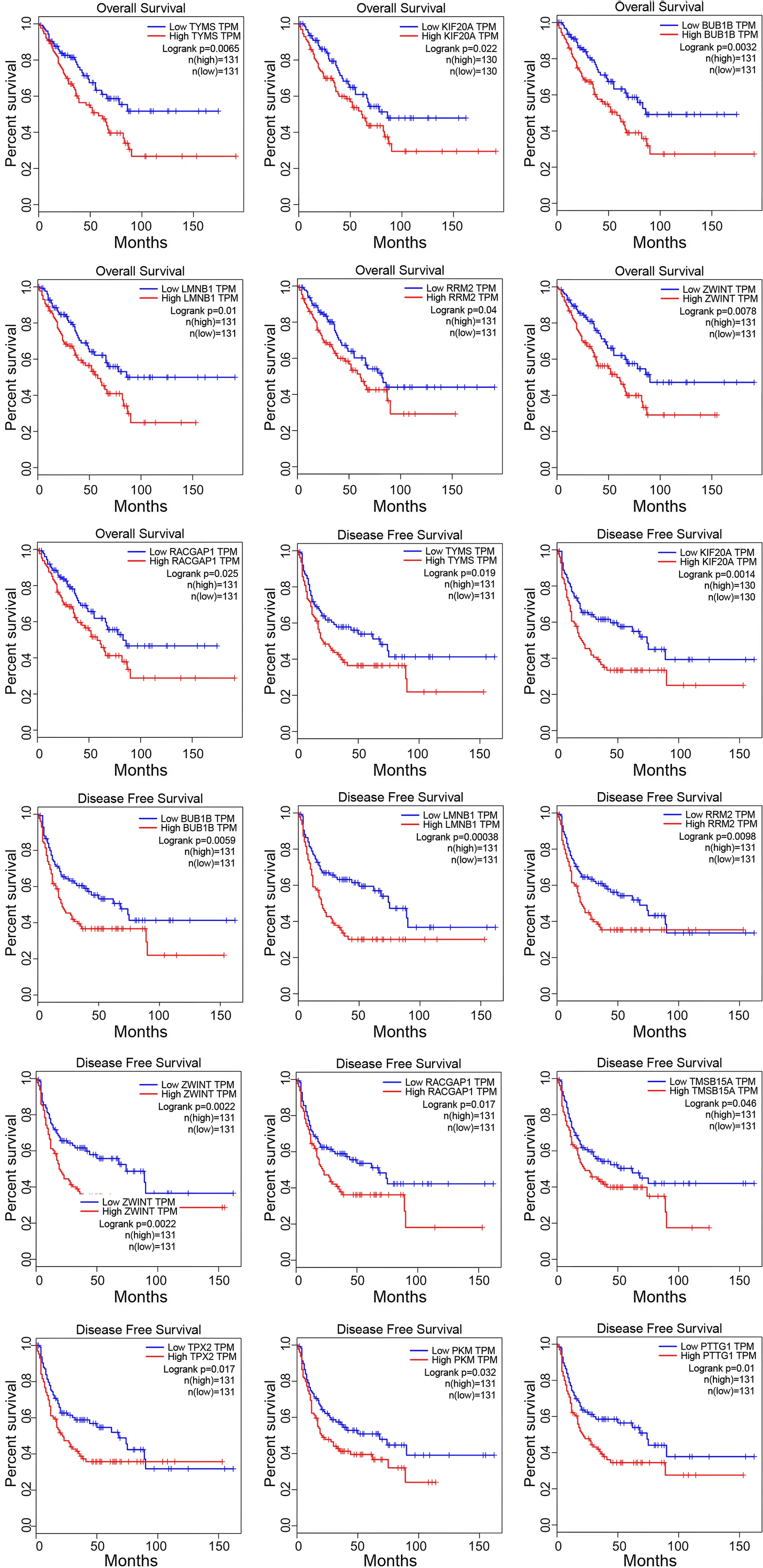
Survival analysis for proliferation-associated genes was performed in sarcoma patients using GEPIA. High expression of 7 genes was significantly associated with both poor overall survival (OS) and poor disease-free survival (DFS), high expression of 4 genes was just significantly associated with poor DFS. (http://gepia.cancer-pku.cn/?from=timeline&isappinstalled=0).

To evaluate whether the pro-proliferative genes identified in our previous cellular studies [8] translate to poor prognosis in sarcoma patients, GEPIA was used to conduct survival analysis. As shown in Fig 7, high TYMS, kinesin family member 20A (KIF20A), BUB1 mitotic checkpoint serine/threonine kinase B (BUB1B), lamin B1 (LMNB1), RRM2, ZW10 interacting kinetochore protein (ZWINT) and Rac GTPase activating protein 1 (RACGAP1) expression were markedly associated with both poor overall survival (OS) and poor disease-free survival (DFS) (OS, P = 0.0065, P = 0.022, P = 0.0032, P = 0.01, P = 0.04, P = 0.0078 and P = 0.025 respectively; DFS, P = 0.019, P = 0.0014, P = 0.0059, P = 0.00038, P = 0.0098, P = 0.0022 and P = 0.017, respectively), and high thymosin beta 15A, TPX2 microtubule nucleation factor, pyruvate kinase M1/2 and PTTG1 regulator of sister chromatid separation, securing expression were just significantly associated with poor DFS (P = 0.046, P = 0.017, P = 0.032 and P = 0.01, respectively). These data showed that both OS and DFS were shorter in sarcoma patients with a high TYMS, KIF20A, BUB1B, LMNB1, RRM2, ZWINT and RACGAP1 expression, so these genes might play crucial roles in the progression of sarcoma patients and be worthy of further exploration.

### Infiltration of immune cells

**Fig 8:**
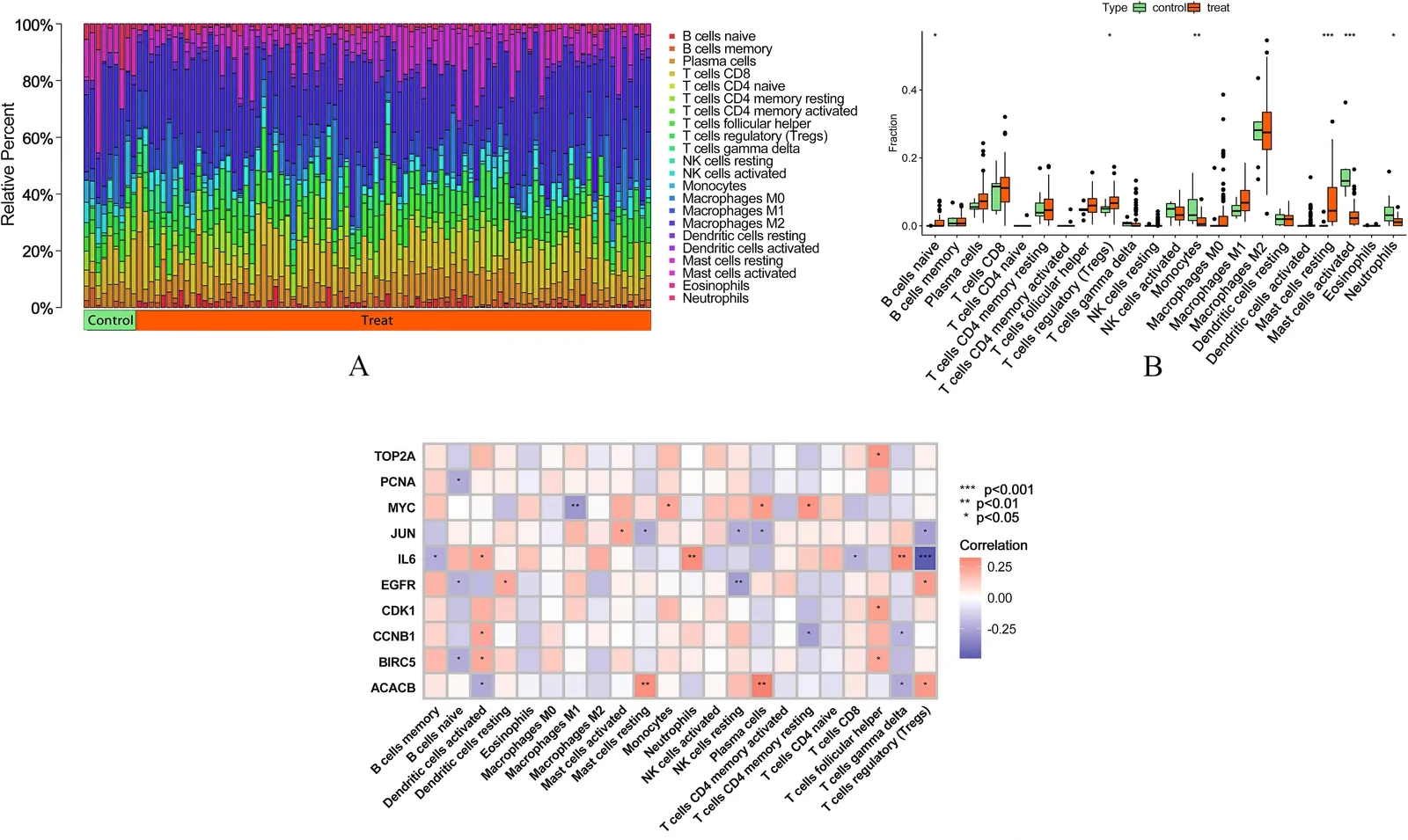
Immune cell composition in LPS and NF tissues. (A) Histogram of the expression levels of 22 types of immune cells in LPS and NF tissues. (B) Differential expressions of immune cell infiltration between LPS and NF tissues. (C) The correlation analysis between hub genes and the 22 cell types identified by CIBERSORT.

The comparative landscape of 22 immune cell populations within LPS and NF is visualized in Fig 8A. In both tissue types, M2 macrophages were the predominant immune component. A notable shift was observed in the LPS microenvironment, which was characterized by an elevated abundance of regulatory T cells (Tregs) alongside a concomitant reduction in activated mast cells (MCs) and neutrophils compared with the NF group. Most other immune subsets demonstrated minimal variation, indicating that these specific differences may warrant further experimental investigation to elucidate their biological significance. As quantitatively detailed in the differential analysis (Fig 8B, the LPS cohort showed a statistically significant decrease (P < 0.05) in activated MCs, monocytes and neutrophils, while exhibiting a significant increase (P < 0.05) in naive B cells, Tregs and resting MCs. Furthermore, the associations between the hub genes and 22 immune cell types were explored (Fig 8C). Correlation analysis identified a significant inverse association between the expression of the hub genes and the infiltration levels of Tregs, M1 macrophages and resting natural killer (NK) cells. Conversely, a positive correlation was observed between hub gene expression and the abundance of neutrophils, resting MCs, plasma cells, and gamma delta T cells.

## Discussion

In the present study, the DEGs were further analyzed. GO and KEGG pathway analysis were subsequently performed, and PPI was constructed to explore the potential genes and pathways associated with the development and progression of LPS. A preliminary test on representative genes was performed using RT-qPCR to verify their differential expression in LPS and NF tissues. In addition, the prognostic significance of genes was assessed in sarcoma patients. Finally, the infiltration of immune cells was analyzed.

Among DEGs, the mRNA expression of genes with a high |log FC| value was examined for preliminary testing in LPS and NF tissues; it was found that RRM2 was highly expressed in retroperitoneal LPS tissues, and patients with high RRM2 expression exhibited significantly shorter OS and DFS. RRM2 has been reported in several types of cancer and been implicated in tumor progression. Xiong *et al* [9] showed that RRM2 modulated immune responses in renal cancer by regulating the annexin A1/AKT signaling axis. Zhuang *et al* [10]found that RRM2 inhibition decreased the Akt phosphorylation level in breast cancer cells, suggesting that RRM2 can regulate the PI3K/Akt pathway, which also exists in LPS cells. Our previous study showed that the mRNA expression of RRM2 was higher in RLPS tissues compared with normal fatty tissues. The downregulation of RRM2 could inhibit cell proliferation, migration and invasion, and promote cell apoptosis. The inhibition of RRM2 could suppress tumor growth in NOD/SCID mice. Along with RRM2 downregulation, the activity of the Akt/mTOR/4EBP1 pathway was also suppressed. These results preliminarily suggested that RRM2 may play a role in the occurrence and progression of LPS; the internal mechanism is therefore worth studying further. Recently, reviews on food and medicine homology have highlighted the potential of bioactive compounds as adjunctive strategies in cancer treatment, providing broader context for the development of novel therapeutic approaches targeting key molecules such as RRM2 [11].

For a deeper understanding of these DEGs, GO terms and KEGG pathway enrichment analysis was performed. The KEGG enrichment analysis of upregulated DEGs showed that the upregulated genes were highly enriched in cell cycle pathway. For instance, PCNA is a protein with cell cycle-dependent properties. In the HT29 colon cancer and FTC236 thyroid cell lines, the p55PIK-PCNA interaction was shown to be involved in regulating DNA synthesis and to contributed to tumorigenesis [12]. Kallakury *et al* [13] noted that in prostate adenocarcinoma, CCNB1 expression was correlated with PCNA (P = 0.0001), and CCNB1 is also a cell proliferation-associated protein. Consistent with these previous studies, the interaction between PCNA and CCNB1 was also revealed through bioinformatics analysis of the PPI network (Fig 6). Similarly, a recent study demonstrated that ferulic acid inhibits colon cancer cell proliferation by inducing cell cycle arrest through the regulation of CDK2 and Cyclin A2, further supporting the importance of cell cycle-related proteins in cancer progression [14]. In addition, a co-expression regulatory network to delineate the interactions among CKS2, RRM2, CDKN2A, MELK, TOP2A, TTK, PLIN1, CIDEC, HBB, CIDEA and PRKAR2B was constructed, and found to be associated with the cell cycle pathway; however, this needs to be further confirmed by molecular experiments.

The common enrichment pathway between upregulated and downregulated genes was HTLV-I infection, which could induce tumorigenesis by activating oncogenes in host cells. HTLV-I infection was the etiological agent of adult T-cell leukemia (ATL) [15]. Akl *et al* [16] indicated that in ATL, HTLV-I infection could activate the Akt/Bad anti-apoptotic pathway, thus providing conditions for malignant transformation.

The PI3K-Akt signaling pathway enriched in module 2 has been widely studied. It regulates crucial cellular functions, including proliferation and metabolism, and plays a critical role in tumorigenesis and survival [17]. Buontempo *et al* [18] demonstrated that cancers activated by PI3K/AKT signaling are more aggressive, and AKT pathway activation has been shown to be an important risk factor for early recurrence and poor prognosis in patients with liver cancer. The negative regulation of the PI3K pathway was controlled by a cluster of lipid phosphatases, among which PTEN was the most representative and clearest [19]. Smith *et al* [20] showed that patients with DDLPS exhibiting a low PTEN expression were sensitive to the PI3K pathway inhibitor rapamycin, resulting in a significantly reduced tumor growth. PTEN was also identified as a downregulated gene in the DEGs analysis, suggesting its potential as a therapeutic target for LPS, particularly DDLPS.

The genes from the PPI network were analyzed, and it was found that TOP2A had the most connections. In breast cancer, TOP2A was frequently amplified, and its amplification had an adverse effect on the prognosis of early breast cancer [21]. Endometrial cancer patients with a high TOP2A expression exhibited a significantly lower OS and DFS [22]. Takako *et al* [23] revealed that TOP2A enhanced the sensitivity of pancreatic ductal adenocarcinoma to anticancer drugs. In prostate cancer, patients whose tumors co-expressed TOP2A and EZH2 exhibited a significant response to the combination therapy of etoposide and an EZH2 inhibitor [24]. EZH2 was also upregulated in LPS in the present study. The biomarker effect of the combination therapy or the biological function of TOP2A and EZH2 in LPS are also worth studying, as they might act as potential biomarkers and targets for molecular target therapy in LPS.

In particular, the amplification of MDM2 and CDK4 are common cytogenetic markers of WDLPS and DDLPS. In the present study, these two genes are upregulated and involved in several pathways, such as the cell cycle, HTLV-I infection and human papillomavirus infection, so they are regarded as promising therapeutic targets. Several clinical trials have been conducted to assess the therapeutic effect of CDK4 or MDM2 inhibitors in the treatment of LPS. In one study, 74 patients with primary or recurrent WDLPS or DDLPS without chemotherapy were treated with MDM2 inhibitor (siremadlin) and CDK4 inhibitor (ribociclib). The recommended expansion dose was 120 mg siremadlin plus 200 mg QD ribociclib every 3 weeks. Among the 74 patients, 3 achieved a partial response and 38 achieved stable disease [25]. In another multicenter phase II study of the combination of ribociclib (CDK4 inhibitor) and everolimus (mTOR inhibitor), 33.3% of DDLPS patients were progression-free at 16 weeks. The combination of riboximab and everolimus demonstrated therapeutic activity in patients with DDLPS and extended stabilization (≥16 weeks), meeting the primary endpoint. It is worth noting that partial responses were observed [26]. Current clinical trials of MDM2 and CDK4 inhibitors have not achieved an ideal rate of objective disease response. The best therapeutic response observed in most patients was disease stabilization. Therefore, in the future application of MDM2 and CDK4 inhibitors it is necessary to more comprehensively understand patients’ agenetic changes in order to screen those beneficiary patients or to consider the combination with other drugs to improve treatment response. Of the total MLPS cases, > 90% carry the characteristic chromosomal translocation t(12;16)(q13;p11), which gives rise to the FUS-DDIT3 fusion oncogene [27]. A study by Zullow *et al* revealed that the FUS-DDIT3 fusion protein interferes with the recruitment and function of the mammalian SWI/SNF (mSWI/SNF or BAF) complex, an ATP-dependent chromatin remodeler, suggesting a potential mechanism underlying the development of MLPS. At the genetic level, PLPS and MPLPS exhibit complex karyotypes and lack specificity [28]. We acknowledge that current batch sequencing analyses based on public databases may mask the specific signals between subtypes. Future studies will include the validation of these markers in independent cohorts of each subtype, and with use single-cell sequencing technology to analyze their cell origin and function at a higher resolution, which will directly address concerns about complexity.

It is well known that immune infiltration and tumor microenvironment (TME) have important effects on tumor progression and prognosis [29]. Therefore, in the present study, the CIBERSORT algorithm was employed to deconvolute the immune cell profiles of the LPS and NF groups. This approach was undertaken to elucidate the potential involvement of specific immune cell subsets in LPS pathogenesis and to systematically investigate their interplay with a panel of centrally relevant genes—namely TOP2A, IL-6, PCNA, CDK1, JUN, MYC, CCNB1, EGFR, ACACB and BIRC5. MCs are immune cells with diverse functions that play a crucial role in cancer. Under the influence of various biological environments, tumor stages and tumor types, MCs exhibit both pro- and anti-tumor effects [30]. For instance, in non-small cell lung cancer (NSCLC), MCs express a large number of molecules that promote tumor progression, such as stem cell factor, histamine, IL-8, fibroblast growth factor-2, vascular endothelial growth factor and platelet-derived growth factor. At the same time, MCs also secrete molecules with dual characteristics, such as TNF-α and TGF-β, which can exert both anti- and pro-tumor effects depending on the specific circumstances [31]. In the present study, the immune cell differential analysis plot showed a significant decrease (P < 0.05) in activated MCs and a significant increase (P < 0.05) in resting MCs in the LPS group compared with the NF group. This finding suggested that MCs have a greater anti-tumor effect in LPS than a pro-tumor effect. In addition, the CIBERSORT analysis revealed a significant positive correlation between the expression of hub genes and resting MCs infiltration. This finding is particularly intriguing given the increasingly recognized role of MCs in the TME.

In the context of LPS, the accumulation of MCs suggests a potential active role in pathogenesis. We hypothesized that MCs may contribute to LPS progression through several mechanisms: i) By secreting growth-promoting factors that synergize with oncogenic pathways driven by genes such as MYC and BIRC5 [32–34]; ii) by fostering an immunosuppressive TME through the release of TGF-β, which may explain the observed negative correlation with anti-tumor immune cells such as Tregs and resting NK cells [35,36]; and iii) by facilitating tissue remodeling and invasion through the release of proteases, potentially aiding processes regulated by EGFR. The exact role (pro- or anti-tumorigenic) of MCs in LPS is likely context-dependent and warrants further investigation [37,38]. Future studies should focus on validating mast cell density in LPS tissue samples using immunohistochemistry and exploring their potential as a therapeutic target, for instance, by using mast cell-stabilizing drugs or c-Kit inhibitors in preclinical models. However, the underlying mechanism requires further investigation. In the present study, the correlation between the identified hub genes and 22 immune cell types was analyzed, and a negative association was identified between Tregs, M1 macrophages and resting NK cells and the hub genes (TOP2A, IL-6, PCNA, CDK1, JUN, MYC, CCNB1, EGFR, ACACB and BIRC5). It was suggested that these alterations in immune cells may affect the expression of hub genes and thus influence the pathogenesis of LPS. The complex association between hub genes and immune cell infiltration needs to be determine by future experiments.

The CIBERSORT analysis in the present study unveiled a complex landscape of mast cell infiltration in LPS, characterized by a predominant proportion of resting MCs and a minor population of activated MCs. The distinct positive correlations of ACACB with resting MCs and JUN with activated MCs offer an intriguing window into the LPS TME. A novel hypothesis was proposed to reconcile these findings: The LPS TME may be generally immunosuppressive and metabolically unique, favoring a resting immune phenotype. The high expression of ACACB, a key mediator of fatty acid oxidation [39], suggests a metabolic preference that could generate a low-inflammatory, energy-efficient milieu lacking the strong stimuli (e.g., IL-33, IgE) required for robust MC activation. Thus, ACACB might indirectly maintain MCs in a resting state, explaining their high abundance and positive correlation. Conversely, the correlation between the oncogene JUN and activated MCs likely reflects rare, localized pro-inflammatory niches within the tumor. In these foci, diminished metabolic restraint and other unknown signals may trigger JUN expression and concomitant mast cell activation. JUN, a stress-responsive transcription factor, and activated MCs, potent secretors of mitogens and proteases, could engage in a paracrine loop that drives focal tumor progression and invasion. This duality—a largely quiescent background punctuated by aggressive inflammatory hubs—may be a key feature of LPS pathogenesis. Future studies using spatial transcriptomics to map the co-localization of JUN high tumor cells, ACABC high tumor cells and mast cell activation markers are crucial to validate this model.

Several limitations of this study should be acknowledged. First, due to the rarity of liposarcoma, the sample size for RT‑qPCR validation (13 LPS vs. 10 normal tissues) was relatively small, further validation in larger independent cohorts is needed. Second, the survival analysis was performed on the combined TCGA‑SARC cohort rather than on individual liposarcoma subtypes, because of the limited sample size for each subtype. Given the biological heterogeneity of liposarcoma, future studies with larger subtype‑specific cohorts are required. Third, the immune infiltration analysis using CIBERSORT on bulk transcriptomic data provides inferential estimates rather than direct measurements of immune cell populations, and the sample size imbalance between tumor (n = 89) and normal (n = 9) tissues may also affect robustness. Immunohistochemistry or other tissue‑based methods are needed for validation, especially for resting mast cells. Finally, this study is primarily a discovery‑oriented bioinformatics screening with preliminary experimental validation, mechanistic and functional experiments are needed in future work to further elucidate the roles of the identified key genes.In conclusion, the present study identified various DEGs that were probably associated with the development and progression of LPS, and immune cell infiltration analysis suggested that resting MCs may play a potential role in the occurrence and development of LPS, although further experimental validation is needed. These findings revealed the potential pathogenesis of LPS and have provided valuable insights for future research. However, further validation is necessary to confirm these results.

## Supporting information

S1 Table Clinical characteristics of liposarcoma patients. (DOCX)

S2 Table Primer sequences used for real-time PCR in RPLPS and NF tissue samples. (DOCX)

S1 File Raw RT-qPCR data. (XLSX)

## References

[1] Song Z, Lu L, Gao Z, Zhou Q, Wang Z, Sun L, et al. Immunotherapy for liposarcoma: emerging opportunities and challenges. Future Oncol. 2022;18(30):3449–61. doi: 10.2217/fon-2021-1549 36214331

[2] Sbaraglia M, Bellan E, Dei Tos AP. The 2020 WHO classification of soft tissue tumours: news and perspectives. Pathologica. 2021;113(2):70–84. doi: 10.32074/1591-951X-213 33179614 PMC8167394

[3] Tyler R, Wanigasooriya K, Taniere P, Almond M, Ford S, Desai A, et al. A review of retroperitoneal liposarcoma genomics. Cancer Treat Rev. 2020;86:102013. doi: 10.1016/j.ctrv.2020.102013 32278233

[4] Schöffski P, Lahmar M, Lucarelli A, Maki RG. Brightline-1: phase II/III trial of the MDM2-p53 antagonist BI 907828 versus doxorubicin in patients with advanced DDLPS. Future Oncol. 2023;19(9):621–9. doi: 10.2217/fon-2022-1291 36987836

[5] Scapa JV, Cloutier JM, Raghavan SS, Peters-Schulze G, Varma S, Charville GW. DDIT3 immunohistochemistry is a useful tool for the diagnosis of myxoid liposarcoma. Am J Surg Pathol. 2021;45(2):230–9. doi: 10.1097/PAS.0000000000001564 32815829 PMC7796975

[6] Assi T, Ngo C, Faron M, Verret B, Lévy A, Honoré C, et al. Systemic therapy in advanced pleomorphic liposarcoma: a comprehensive review. Curr Treat Options Oncol. 2023;24(11):1598–613. doi: 10.1007/s11864-023-01139-3 37843627

[7] de Bree E, Michelakis D, Heretis I, Kontopodis N, Spanakis K, Lagoudaki E. Retroperitoneal soft tissue sarcoma: emerging therapeutic strategies. Cancers (Basel). 2023;15(22):Epub. doi: 10.3390/cancers15225469 38001729 PMC10670057

[8] Zhang S, Yan L, Cui C, Wang Z, Wu J, Zhao M, et al. Identification of TYMS as a promoting factor of retroperitoneal liposarcoma progression: Bioinformatics analysis and biological evidence. Oncol Rep. 2020;44(2):565–76. doi: 10.3892/or.2020.7635 32627015 PMC7336505

[9] Xiong W, Zhang B, Yu H, Zhu L, Yi L, Jin X. RRM2 regulates sensitivity to sunitinib and PD-1 blockade in renal cancer by stabilizing ANXA1 and activating the AKT pathway. Adv Sci (Weinh). 2021;8(18):e2100881. doi: 10.1002/advs.202100881 34319001 PMC8456228

[10] Cui L, Yan L, Guan X, Dong B, Zhao M, Lv A, et al. Anti-tumor effect of apatinib and relevant mechanisms in liposarcoma. Front Oncol. 2021;11:739139. doi: 10.3389/fonc.2021.739139 34868934 PMC8637299

[11] Li M-Y, Zhang Q, Li J, Zengin G. Food and medicine homology in cancer treatment: traditional thoughts collide with scientific evidence. Food Med Homol. 2025;2(3):9420120. doi: 10.26599/fmh.2025.9420120

[12] Wang G, Cao X, Lai S, Luo X, Feng Y, Xia X, et al. PI3K stimulates DNA synthesis and cell-cycle progression via its p55PIK regulatory subunit interaction with PCNA. Mol Cancer Ther. 2013;12(10):2100–9. doi: 10.1158/1535-7163.MCT-12-0920 23939377

[13] Kallakury BVS, Sheehan CE, Rhee SJ, Fisher HAG, Kaufman RP, Rifkin MD, et al. The prognostic significance of proliferation-associated nucleolar protein p120 expression in prostate adenocarcinoma. Cancer. 1999;85(7):1569–76. doi: 10.1002/(sici)1097-0142(19990401)85:7<1569::aid-cncr19>3.0.co;2-m 10193948

[14] Liu Z-P, Tang W-S, Wang G-Z, Xu J-W, Zhu L-J, Lyu Q-Y, et al. Ferulic acid inhibiting colon cancer cells at different Duke’s stages. Food Med Homol. 2025;2(3):9420063. doi: 10.26599/fmh.2025.9420063

[15] Bellon M, Nicot C. Feedback loop regulation between Pim kinases and tax keeps human t-cell leukemia virus type 1 viral replication in check. J Virol. 2022;96(3):e0196021. doi: 10.1128/JVI.01960-21 34818069 PMC8826812

[16] Akl H, Badran BM, Zein NE, Bex F, Sotiriou C, Willard-Gallo KE, et al. HTLV-I infection of WE17/10 CD4+ cell line leads to progressive alteration of Ca2+ influx that eventually results in loss of CD7 expression and activation of an antiapoptotic pathway involving AKT and BAD which paves the way for malignant transformation. Leukemia. 2007;21(4):788–96. doi: 10.1038/sj.leu.2404585 17287851

[17] Glaviano A, Foo ASC, Lam HY, Yap KCH, Jacot W, Jones RH, et al. PI3K/AKT/mTOR signaling transduction pathway and targeted therapies in cancer. Mol Cancer. 2023;22(1):138. doi: 10.1186/s12943-023-01827-6 37596643 PMC10436543

[18] Tian L-Y, Smit DJ, Jücker M. The role of PI3K/AKT/mTOR signaling in hepatocellular carcinoma metabolism. Int J Mol Sci. 2023;24(3):2652. doi: 10.3390/ijms24032652 36768977 PMC9916527

[19] Chessa TAM, Jung P, Anwar A, Suire S, Anderson KE, Barneda D, et al. PLEKHS1 drives PI3Ks and remodels pathway homeostasis in PTEN-null prostate. Mol Cell. 2023;83(16):2991-3009.e13. doi: 10.1016/j.molcel.2023.07.015 37567175

[20] Stefano S, Giovanni S. The PTEN tumor suppressor gene in soft tissue sarcoma. Cancers (Basel). 2019;11(8):1169. doi: 10.3390/cancers11081169 31416195 PMC6721622

[21] Mehraj U, Qayoom H, Shafi S, Farhana P, Asdaq SMB, Mir MA. Cryptolepine targets TOP2A and inhibits tumor cell proliferation in breast cancer cells - an in vitro and in silico study. Anticancer Agents Med Chem. 2022;22(17):3025–37. doi: 10.2174/1871520622666220419135547 35440335

[22] Ito F, Furukawa N, Nakai T. Evaluation of TOP2A as a predictive marker for endometrial cancer with taxane-containing adjuvant chemotherapy. Int J Gynecol Cancer. 2016;26(2):325–30. doi: 10.1097/IGC.0000000000000607 26588239

[23] Tanaka T, Okada R, Hozaka Y, Wada M, Moriya S, Satake S, et al. Molecular pathogenesis of pancreatic ductal adenocarcinoma: impact of miR-30c-5p and miR-30c-2-3p regulation on oncogenic genes. Cancers (Basel). 2020;12(10). doi: 10.3390/cancers12102731 32977589 PMC7598296

[24] Labbé DP, Sweeney CJ, Brown M, Galbo P, Rosario S, Wadosky KM, et al. TOP2A and EZH2 provide early detection of an aggressive prostate cancer subgroup. Clin Cancer Res. 2017;23(22):7072–83. doi: 10.1158/1078-0432.CCR-17-0413 28899973 PMC5690819

[25] Abdul Razak AR, Bauer S, Suarez C, Lin C-C, Quek R, Hütter-Krönke ML, et al. Co-targeting of MDM2 and CDK4/6 with siremadlin and ribociclib for the treatment of patients with well-differentiated or dedifferentiated liposarcoma: results from a proof-of-concept, phase ib study. Clin Cancer Res. 2022;28(6):1087–97. doi: 10.1158/1078-0432.CCR-21-1291 34921024

[26] Movva S, Matloob S, Handorf EA, Choy E, Merriam P, Flieder DB, et al. SAR-096: phase II clinical trial of ribociclib in combination with everolimus in advanced dedifferentiated liposarcoma (DDL) and leiomyosarcoma (LMS). Clin Cancer Res. 2024;30(2):315–22. doi: 10.1158/1078-0432.CCR-23-2469 37967116

[27] Craparotta I, Mannarino L, Zadro R, Ballabio S, Marchini S, Pavesi G, et al. Mechanism of efficacy of trabectedin against myxoid liposarcoma entails detachment of the FUS-DDIT3 transcription factor from its DNA binding sites. J Exp Clin Cancer Res. 2024;43(1):309. doi: 10.1186/s13046-024-03228-z 39587691 PMC11590625

[28] Zhou X-P, Xing J-P, Sun L-B, Tian S-Q, Luo R, Liu W-H, et al. Molecular characteristics and systemic treatment options of liposarcoma: a systematic review. Biomed Pharmacother. 2024;178:117204. doi: 10.1016/j.biopha.2024.117204 39067161

[29] Tang R, Liu X, Liang C, Hua J, Xu J, Wang W, et al. Deciphering the prognostic implications of the components and signatures in the immune microenvironment of pancreatic ductal adenocarcinoma. Front Immunol. 2021;12:648917. doi: 10.3389/fimmu.2021.648917 33777046 PMC7987951

[30] Valent P, Akin C, Hartmann K, Nilsson G, Reiter A, Hermine O, et al. Mast cells as a unique hematopoietic lineage and cell system: from Paul Ehrlich’s visions to precision medicine concepts. Theranostics. 2020;10(23):10743–68. doi: 10.7150/thno.46719 32929378 PMC7482799

[31] Longo V, Catino A, Montrone Mi, Galetta D, Ribatti D. Controversial role of mast cells in NSCLC tumor progression and angiogenesis. Thorac Cancer. 2022;13(21):2929–34. doi: 10.1111/1759-7714.14654 36196487 PMC9626321

[32] Maltby S, Khazaie K, McNagny KM. Mast cells in tumor growth: angiogenesis, tissue remodelling and immune-modulation. Biochim Biophys Acta. 2009;1796(1):19–26. doi: 10.1016/j.bbcan.2009.02.001 19233249 PMC2731828

[33] Ribatti D, Crivellato E. The controversial role of mast cells in tumor growth. Int Rev Cell Mol Biol. 2009;275:89–131. doi: 10.1016/S1937-6448(09)75004-X 19491054

[34] Khazaie K, Blatner NR, Khan MW, Gounari F, Gounaris E, Dennis K, et al. The significant role of mast cells in cancer. Cancer Metastasis Rev. 2011;30(1):45–60. doi: 10.1007/s10555-011-9286-z 21287360

[35] Gri G, Frossi B, D’Inca F, Danelli L, Betto E, Mion F, et al. Mast cell: an emerging partner in immune interaction. Front Immunol. 2012;3:120. doi: 10.3389/fimmu.2012.00120 22654879 PMC3360165

[36] Huang B, Lei Z, Zhang G-M, Li D, Song C, Li B, et al. SCF-mediated mast cell infiltration and activation exacerbate the inflammation and immunosuppression in tumor microenvironment. Blood. 2008;112(4):1269–79. doi: 10.1182/blood-2008-03-147033 18524989 PMC2515142

[37] Coussens LM, Raymond WW, Bergers G, Laig-Webster M, Behrendtsen O, Werb Z, et al. Inflammatory mast cells up-regulate angiogenesis during squamous epithelial carcinogenesis. Genes Dev. 1999;13(11):1382–97. doi: 10.1101/gad.13.11.1382 10364156 PMC316772

[38] Komi DEA, Redegeld FA. Role of mast cells in shaping the tumor microenvironment. Clin Rev Allergy Immunol. 2020;58(3):313–25. doi: 10.1007/s12016-019-08753-w 31256327 PMC7244463

[39] Hong H-J, Shao Y, Zhang S, Yang G, Jia H, Yang X, et al. ACACB is a novel metabolism-related biomarker in the prediction of response to cetuximab therapy inmetastatic colorectal cancer. Acta Biochim Biophys Sin (Shanghai). 2022;54(11):1671–83. doi: 10.3724/abbs.2022121 36111743 PMC9828296

