# Supplementary material for: Identification of functional genes and immune cell infiltration analysis in liposarcoma
Source: PLoS One. 2026 Sep 11;21(9):e0358180. doi: 10.1371/journal.pone.0358180 (PMC13568520; doi:10.1371/journal.pone.0358180)
Supplement: Supplementary file 1 [file pone.0358180.s001.docx]

| S1 Table. Clinical characteristics of liposarcoma patients. | | | | |
| --- | --- | --- | --- | --- |
| Sample ID | Sex | Age | Subtype | Grade |
| LPS-01 | F | 33 | WDLPS | G2 |
| LPS-02 | F | 57 | MLPS | G3 |
| LPS-03 | M | 68 | MLPS | G3 |
| LPS-04 | M | 68 | DDLPS | G3 |
| LPS-05 | M | 61 | WDLPS | G2 |
| LPS-06 | F | 53 | DDLPS | G3 |
| LPS-07 | M | 62 | DDLPS | G3 |
| LPS-08 | M | 57 | MLPS | G3 |
| LPS-09 | F | 65 | WDLPS | G2 |
| LPS-10 | M | 60 | WDLPS | G2 |
| LPS-11 | M | 55 | DDLPS | G3 |
| LPS-12 | F | 62 | WDLPS | G2 |
| LPS-13 | M | 53 | PLPS | G3 |
| WDLPS：Well‑differentiated liposarcoma | | | | |
| DDLPS：Dedifferentiated liposarcoma | | | | |
| MLPS：Myxoid liposarcoma | | | | |
| PLPS：Pleomorphic liposarcoma | | | | |

F: Female

M: Male
