# Supplementary material for: Identification of functional genes and immune cell infiltration analysis in liposarcoma
Source: PLoS One. 2026 Sep 11;21(9):e0358180. doi: 10.1371/journal.pone.0358180 (PMC13568520; doi:10.1371/journal.pone.0358180)
Supplement: Supplementary file 2 [file pone.0358180.s002.docx]

| **S2 Table.** Primer sequences used for real-time PCR in RPLPS and NF tissue samples. | | |
| --- | --- | --- |
| GAPDH | F: 5’-TGACTTCAACAGCGACACCCA-3’ | R: 5’-CACCCTGTTGCTGTAGCCAAA-3’ |
| CKS2 | F: 5’-TTCGACGAACACTACGAGTACC-3’ | R: 5’-GGACACCAAGTCTCCTCCAC-3’ |
| CCNB1 | F: 5’-TATCTGAGACAACTTGAGGAAG-3’ | R: 5’-ACTAGCCAGTCAATTAGGATG-3’ |
| CDKN2A | F: 5’-GCCCAACGCACCGAATAGTT-3’ | R: 5’-CACGGGTCGGGTGAGAGT-3’ |
| CIDEA | F: 5’-TTCCGGGTCTCCAACCATGA-3’ | R: 5’- CCAGAGTGACCAGTCCGGTA-3’ |
| RRM2 | F: 5’-AAGAAACGAGGACTGATGC-3’ | R: 5’-CTGTCTGCCACAAACTCAA-3’ |
| CIDEC | F: 5’-AACGCAGTCCAGCTGACAAG-3’ | R: 5’-TGGGTCACCACAGAGGTACG-3’ |
| HBB | F: 5’-AGAAGTCTGCCGTTACTGCC-3’ | R: 5’-GTCCAAGGGTAGACCACCAG-3’ |
| PCNA | F: 5’-GCCAGAGCTCTTCCCTTAC-3’ | R: 5’-TAGCTGGTTTCGGCTTCAGG-3’ |
| PLIN1 | F: 5’-CTGGAGGAAAAGATCCCCGC-3’ | R: 5’-AGATGGTGTCCTTCAGCTCAG-3’ |
| PPP1R1A | F: 5’-CCCCCGAAAGATCCAGTTCA-3’ | R: 5’-ATGACTGGTCACTGGTCAGC-3’ |
| PRKAR2B | F: 5’-AAACCGATTCACAAGGCGTG-3’ | R: 5’-CCTGGACTCTGCATCATCTTCT-3’ |
| SAA1 | F: 5’-GGGGAACTATGATGCTGCCA-3’ | R: 5’-GCACCATGGCCAAAGAATCTC-3’ |
| TOP2A | F: 5’-CCGTCACCATGGAAGTGTCA-3’ | R: 5’-GACAGTCTTTTCTTAGCATCTTCAT-3’ |
| TTK | F: 5’-CTTTTCATTTCCCCAGCGCA-3’ | R: 5’-TCTCTGCCACTTAAATCCTCGG-3’ |
| ZIC1 | F: 5’-CAAGATGTGCGACAAGTC-3’ | R: 5’-GAGGATTCGTAGCCAGAG-3’ |
| MELK | F: 5’-TATTCACCTCGATGATGATTGCG-3’ | R: 5’-AGAAAGCCTTAAACGAACTGGTT-3’ |
