# Supplementary material for: Identification of functional genes and immune cell infiltration analysis in liposarcoma
Source: PLoS One. 2026 Sep 11;21(9):e0358180. doi: 10.1371/journal.pone.0358180 (PMC13568520; doi:10.1371/journal.pone.0358180)
Supplement: Supplementary file 3 [file pone.0358180.s005.docx]

Dear Editor and Reviewers,

We would like to express our sincere gratitude to the Academic Editor and the reviewers for their careful reading and thoughtful comments on our manuscript (PONE-D-26-29480) entitled “Identification of functional genes and immune cell infiltration analysis in liposarcoma.” We greatly appreciate the time and effort dedicated to evaluating our work.

We have carefully considered all the comments and have revised the manuscript accordingly. Below we provide our point-by-point responses to each of the reviewers’ comments. All changes in the revised manuscript have been highlighted using the track changes function in Microsoft Word, and we have provided a clean version without tracked changes for your convenience.

**A note to the Editor**

We would like to respectfully bring to the Editor's attention that the comments provided for Reviewer #2 appear to pertain to a different manuscript. The reviewer's feedback discusses topics such as "diabetic retinopathy," "hypoxia," and "cuproptosis," and references genes (e.g., MED19, CA11) and datasets (e.g., GSE189005) that are not part of our study on liposarcoma. We would be grateful if the editorial office could clarify this matter or, if necessary, arrange for an additional review. We remain available to provide any further information required.

We believe that the revised manuscript has been substantially improved and hope it is now suitable for publication in PLOS ONE.”

We respond to each point raised below in bold, with our responses in plain text.

**Response to Journal Requirements:**

**1. Please ensure that your manuscript meets PLOS ONE's style requirements, including those for file naming.**

We thank the editor for this reminder. We have carefully revised the manuscript to comply with PLOS ONE's style requirements. The specific changes we have made include:

Manuscript body formatting: We have reformatted all section headings according to the PLOS ONE guidelines. Level 1 headings (Abstract, Introduction, Materials and methods, Results, Discussion, etc.) are now set to bold, 18pt; Level 2 headings are set to bold, 16pt. All headings have been revised to sentence case (only the first word and proper nouns are capitalized). The entire manuscript is now formatted with double-spacing as required.

Figure citations and captions: All figure citations have been revised to follow the "Fig 1", "Fig 2" format and are cited in the correct numerical order. Figure captions have been placed directly after the paragraph in which each figure is first cited, with the figure titles in bold.

Reference citations: All references are now cited using the correct format and are listed in the order of first appearance in the text.

File naming: All uploaded files have been renamed according to PLOS ONE's file naming requirements, using only letters, numbers, and underscores.

Supporting Information: The Supporting Information files have been labeled as "S1 Table", "S2 Table" and "S1 File" are listed in the manuscript with the correct format.

We believe the manuscript now fully complies with PLOS ONE's formatting and file naming requirements.

**2. Please note that PLOS ONE has specific guidelines on code sharing for submissions in which author-generated code underpins the findings in the manuscript.**

We thank the editor for this reminder. We have reviewed the PLOS ONE guidelines on code sharing. In our study, we used author-generated R scripts for data preprocessing, GO and KEGG analysis, and immune cell infiltration estimation (CIBERSORT). To comply with the policy, we have deposited all custom R code in a public repository figshare and have included the repository link and DOI in the Data Availability Statement. The repository link is: https://doi.org/10.6084/m9.figshare.33182657. The repository link has also been included in the Data Availability Statement in the revised manuscript.

**3. We note that the grant information you provided in the ‘Funding Information’ and ‘Financial Disclosure’ sections do not match. When you resubmit, please ensure that you provide the correct grant numbers for the awards you received for your study in the ‘Funding Information’ section.**

We thank the editor for pointing out this discrepancy. We have corrected the funding information in the submission system. Both the ‘Funding Information’ section in the system and the financial disclosure now consistently list the following grant numbers: Shandong Provincial Natural Science Foundation (Grant Nos. ZR2021QH260 and ZR2021QH344). Additionally, as per the journal's requirement that funding information should not appear in the manuscript body, we have removed all funding-related text from the manuscript. The funding information now appears only in the online submission system and in the cover letter (where we have included the role of funders statement as requested). We confirm that the online system and the cover letter are now fully aligned.

**4. Thank you for stating the following financial disclosure: “This study was supported by Shandong Provincial Natural Science Foundation (approval No. ZR2021QH260 and approval No. ZR2021QH344).” Please state what role the funders took in the study. If the funders had no role, please state: "The funders had no role in study design, data collection and analysis, decision to publish, or preparation of the manuscript." If this statement is not correct you must amend it as needed. Please include this amended Role of Funder statement in your cover letter; we will change the online submission form on your behalf.**

We thank the editor for this reminder. As requested, we have now included the following Role of Funder statement in the cover letter: "The funders had no role in study design, data collection and analysis, decision to publish, or preparation of the manuscript." We confirm that this statement accurately reflects the funders' role in our study. We also confirm that the funders had no additional involvement beyond the financial support provided.

**5. Please note that funding information should not appear in any section or other areas of your manuscript. We will only publish funding information present in the Funding Statement section of the online submission form. Please remove any funding-related text from the manuscript.**

We thank the editor for this reminder. We have carefully reviewed the entire manuscript and have removed all funding-related text from all sections, including the title page, abstract, acknowledgments, and any other areas of the manuscript. Funding information now appears only in the Funding Statement section of the online submission system, consistent with the journal's policy. The corresponding Role of Funder statement has been included in the cover letter as requested.

**6. Your ethics statement should only appear in the Methods section of your manuscript. If your ethics statement is written in any section besides the Methods, please delete it from any other section.**

We thank the editor for this reminder. We have carefully reviewed the entire manuscript and confirm that the ethics statement now appears only in the Methods section. Any ethics-related text that was previously present in other sections has been deleted. The ethics statement in the Methods section includes the approval number, approval date, and a statement that the study was conducted in accordance with the Declaration of Helsinki, as shown below: "This study was approved by the Ethics Committee of Shandong Provincial Hospital for Biomedical Research Involving Human Subjects (Approval No. SZRJJ:NO.2021-037) on March 1, 2021. All human tissue samples were collected between March 1, 2021 and March 1, 2022. Written informed consent was obtained from all participants prior to sample collection. The study was conducted in accordance with the Declaration of Helsinki."

**7. Please include your tables as part of your main manuscript and remove the individual files. Please note that supplementary tables (should remain/ be uploaded) as separate "supporting information" files.**

We thank the editor for this reminder. We have now embedded all main tables directly into the main manuscript file. Each table has been placed after the paragraph in which it is first cited. The individual table files have been removed from the submission system. In addition, we have two supplementary tables (S1 Table, S2 Table and S1 File), which have been uploaded as a separate "Supporting Information" file. These tables have also been listed in the Supporting Information section at the end of the manuscript.

**8. Please include captions for your Supporting Information files at the end of your manuscript, and update any in-text citations to match accordingly. Please see our Supporting Information guidelines for more information:** [**http://journals.plos.org/plosone/s/supporting-information**](http://journals.plos.org/plosone/s/supporting-information)**.**

We thank the editor for this reminder. We have now added a "Supporting Information" section at the end of the manuscript (after the References) with the following captions for all supporting files: S1 and S2 Table. Clinical characteristics of liposarcoma patients and Primer sequences used for real-time PCR in RPLPS and NF tissue samples; S1 File. Raw RT-qPCR data. We have also reviewed the manuscript and confirm that all in-text citations to these supporting files now use the correct format (S1 Table, S2 Table and S1 File) and are listed in the Supporting Information section accordingly.

**9. Thank you for providing your underlying data as Supporting Information. We note that the data set (primer.zip) contains text or data that is not in English. Please note that PLOS is an English-language publisher, so we require data sets to be provided in English as well. Please upload an English-language version of your data set. This will also allow us to determine if your data follows PLOS standards per our Data Availability policy here:** [**https://journals.plos.org/plosone/s/data-availability**](https://journals.plos.org/plosone/s/data-availability)**.**

We thank the editor for this reminder. After carefully reviewing the files we initially submitted, we noted that the content of primer.zip was redundant with the information already provided in the Supporting Information files (S2 Table). To avoid duplication and ensure clarity, we have decided not to re-upload primer.zip in the revised submission. We have also updated the Data Availability Statement in the manuscript to clearly indicate that the RT-qPCR data are available in S2 Table and S1 File, and the primer.zip file has been removed from the submission system.

**10. If the reviewer comments include a recommendation to cite specific previously published works, please review and evaluate these publications to determine whether they are relevant and should be cited. There is no requirement to cite these works unless the editor has indicated otherwise.**

We thank the editor for this reminder. We have carefully reviewed the publications recommended by Reviewer #1. Please see our detailed response to Reviewer #1, Comment 7 below, where we explain which papers we have cited and our reasons for doing so.

**Response to Reviewer #1:**

1. **Comment 1: Several methodological details should be clarified to improve reproducibility. The authors state that the data processing and differential expression analysis followed a previous study, but the present manuscript should still provide sufficient methodological information, including the normalization method, log transformation, probe annotation, duplicate probe handling, statistical model, and multiple-testing correction.**

**Response:** We thank the reviewer for this important suggestion. We agree that methodological details should be described within the current manuscript rather than relying solely on a previous reference. In the revised manuscript, we have added a brief description of the differential expression analysis in the Methods section (Lines 70-74), including the normalization method, statistical model, and multiple-testing correction. This description is concise and appropriately references our previous publication for the full details. We believe this approach provides sufficient methodological transparency while maintaining the independence of the current study.

2. **Comment 2: The criteria used to define DEGs, hub genes, diagnostic biomarkers, and RT-qPCR validation candidates should be presented more clearly, because different gene lists appear in different sections of the manuscript.**

**Response:** We agree with the reviewer that the criteria for defining different gene lists should be clearly stated. In the revised manuscript, we have added a dedicated subsection in the Methods section that explicitly defines each category as follows: Differentially expressed genes (DEGs): Genes with |log2FC| > 1 and FDR < 0.05, identified by the limma package comparing liposarcoma tissues versus normal fatty tissues (Lines 70-74). Hub genes: The top 10 genes ranked by node degree in the protein-protein interaction (PPI) network constructed using the STRING database and visualized with Cytoscape. Node degree reflects the number of connections a gene has with other genes in the network (Lines 83-85). Diagnostic biomarkers: The 10 hub genes identified from the PPI network, which were considered as potential diagnostic biomarkers for liposarcoma based on their central positions in the network and their differential expression patterns (Lines 203-205). RT-qPCR validation candidates: Criteria added to the Methods section, “RT-qPCR validation” subsection. Candidate genes were selected based on both high |log₂FC| values and documented biological significance in cancer or liposarcoma from previous literature (Lines 99-101). We believe this clarification makes the relationship between the different gene lists transparent and improves the overall readability of the manuscript.

3. **The survival analysis also requires additional clarification. The authors should specify the source of survival data, the number of liposarcoma cases included, the survival endpoints used, and whether different histological subtypes of liposarcoma were analyzed together or separately. Since liposarcoma is biologically heterogeneous, subtype composition may influence gene expression and survival associations. If subtype-specific analysis is not feasible, this issue should be discussed as a limitation.**

**Response:** We thank the reviewer for this important suggestion. In the revised manuscript, we have now provided the following clarifications regarding the survival analysis: Data source: We have clarified that the survival analysis was performed using the GEPIA online platform, which is based on RNA-seq data from The Cancer Genome Atlas (TCGA) and genotype-tissue expression (GTEx) projects (Lines 115-118). Number of cases: The analysis included 263 sarcoma patients from the TCGA-SARC cohort (Lines 118-120). We acknowledge that this cohort includes multiple sarcoma subtypes rather than liposarcoma alone, as this was the only sarcoma cohort available with sufficient sample size for survival analysis in the GEPIA database. Survival endpoints: Overall survival (OS) and disease-free survival (DFS) were used as the endpoints (Lines 122-123). Subtype analysis: Due to the limited sample size of individual liposarcoma subtypes within the TCGA-SARC cohort, subtype‑specific survival analysis was not feasible. Therefore, the analysis was performed on the combined sarcoma cohort. As suggested by the reviewer, we have acknowledged this as a limitation in the Discussion section (Lines 464-467). We believe these additions address the reviewer's concerns and provide sufficient transparency regarding the survival analysis.

4. **The immune infiltration analysis is interesting, but the interpretation should be more cautious. CIBERSORT results based on bulk transcriptomic data are inferential and may be affected by sample size imbalance between tumor and normal fatty tissues. The authors should report the CIBERSORT P-value filtering strategy, whether multiple comparisons were corrected, and whether immune-cell findings were validated by independent datasets or tissue markers. The conclusion that resting mast cells are involved in liposarcoma development should be softened unless supported by additional experimental evidence.**

**Response:** We thank the reviewer for this important and constructive suggestion. We agree that CIBERSORT-based immune infiltration results should be interpreted with caution. In the revised manuscript, we have made the following changes: CIBERSORT methodology: In the Methods section (Lines 129-132), we have now specified that the analysis was performed with 1,000 permutations and that only samples with CIBERSORT P < 0.05 were retained. We also note that no multiple comparison correction was applied, as this was an exploratory analysis, and we acknowledge this as a limitation in the Discussion section. Interpretation of immune infiltration findings: In the Discussion section (Lines 468-472), we have added a discussion on the inferential nature of CIBERSORT results and the limitations associated with the sample size imbalance in the GSE21122 dataset. We also note the absence of independent validation by tissue-based methods (such as immunohistochemistry) and emphasize the need for future validation studies. Softened conclusions: We have revised the conclusions regarding resting mast cells throughout the manuscript. Specifically, in the Abstract and Discussion, we have changed the wording from "resting MCs were significantly involved in the occurrence and development of LPS" to a more cautious interpretation, such as "resting MCs may play a potential role in LPS pathogenesis," and emphasize that these findings are exploratory and require further experimental validation. We believe these revisions address the reviewer's concerns and ensure that our conclusions are appropriately supported by the data presented.

5. **The RT-qPCR validation strengthens the manuscript, but more clinical and technical information is needed. The authors should provide patient characteristics, tissue source, liposarcoma subtype, tumor grade if available, primer sequences, housekeeping gene, and the statistical method used for qPCR analysis.**

**Response:** We thank the reviewer for this suggestion. In the revised manuscript, we have now provided the following information regarding the RT-qPCR validation. Patient characteristics, tissue source, liposarcoma subtype, and tumor grade: We have added a summary table (S1 Table) as Supporting Information, which includes patient age, sex, tissue source, liposarcoma subtype, and tumor grade. This information is also briefly summarized in the Methods section (Lines 90-91). Primer sequences: All primer sequences (forward and reverse) are now provided in S2 Table. Housekeeping gene: GAPDH was used as the internal reference gene. This has been stated in the Methods section (Lines 109-110). Statistical method for RT-qPCR analysis: The 2^(-ΔΔCt) method was used for relative quantification. Statistical comparisons between LPS and normal fatty tissues were performed using the independent samples t-test. This has been added to the Statistical analysis section (Lines 137-139). We believe these additions provide sufficient clinical and technical details for readers to evaluate the RT-qPCR validation.

6. **Figure quality and legends should also be improved so that gene names, statistical annotations, and group labels are clearly readable.**

**Response:** We thank the reviewer for this suggestion. We have regenerated all figures at high resolution in TIFF format to ensure optimal clarity. All gene names, statistical annotations and group labels have been enlarged and reformatted for improved readability. In addition, multi-panel figures have been combined into single image files as required. All revised figures have been submitted separately and have passed the quality check using the PLOS-recommended NAAS tool. The figure legends have also been reviewed and updated to ensure they clearly describe all content and abbreviations in each figure.

7. **Since the present manuscript identifies cell-cycle-related hub genes and survival-related biomarkers in liposarcoma, “Ferulic acid inhibiting colon cancer cells at different Duke’s stages” (DOI: 10.26599/FMH.2025.9420063) is relevant because it involves cancer-cell inhibition, apoptosis, and cell-cycle regulatory proteins. The review “Food and medicine homology in cancer treatment: traditional thoughts collide with scientific evidence” (DOI: 10.26599/FMH.2025.9420120) may also provide broader background for discussing bioactive compounds and cancer treatment strategies. If the authors expand the discussion toward future targeted intervention based on hub genes, “Research progress of microrobots in tumor drug delivery” (DOI: 10.26599/FMH.2024.9420025) would be relevant as it focuses on tumor drug-delivery strategies.**

**Response:** We thank the reviewer for these valuable literature recommendations. We have carefully reviewed all three publications and have incorporated the relevant ones into our revised manuscript:

Ferulic acid inhibiting colon cancer cells at different Duke’s stages (DOI: 10.26599/FMH.2025.9420063) — We agree with the reviewer that this paper is relevant to our study, as it discusses cell-cycle regulatory proteins and apoptosis in cancer. We have now cited it in the Discussion section (Lines 341-343) to provide broader context for our cell-cycle-related hub gene findings.

Food and medicine homology in cancer treatment: traditional thoughts collide with scientific evidence (DOI: 10.26599/FMH.2025.9420120) — This review provides useful background on cancer treatment strategies and bioactive compounds. We have cited it in the Discussion section (Lines 328-331).

Research progress of microrobots in tumor drug delivery (DOI: 10.26599/FMH.2024.9420025) — While this paper is interesting and relevant to drug delivery strategies, our current study focuses on gene expression and immune infiltration in liposarcoma rather than targeted drug delivery approaches. Therefore, we have not cited it in this revision, as it falls outside the main scope of our discussion.

We appreciate the reviewer’s suggestions, which have helped us strengthen the contextual background of our study.

**Response to Reviewer #2:**

We respectfully refer the Editor to our note at the beginning of this letter regarding the comments assigned to Reviewer #2. As these comments pertain to a different manuscript and are not applicable to our study, we are unable to address them point-by-point. We would be grateful if the editorial office could clarify this matter or arrange for an additional review if necessary.
