# Supplementary material for: Identification of functional genes and immune cell infiltration analysis in liposarcoma
Source: PLoS One. 2026 Sep 11;21(9):e0358180. doi: 10.1371/journal.pone.0358180 (PMC13568520; doi:10.1371/journal.pone.0358180)
Supplement: Peer review history

# Peer review: Decision Letter 0

**DOI:** 10.1371/journal.pone.0358180.r001

**Authors:** Kangkang Ji

14 Jul 2026

Dear Dr. Zhang,

Thank you for submitting your manuscript to PLOS ONE. After careful consideration, we feel that it has merit but does not fully meet PLOS ONE’s publication criteria as it currently stands. Therefore, we invite you to submit a revised version of the manuscript that addresses the points raised during the review process.

Please submit your revised manuscript by Sep 12 2026 11:59PM. If you will need more time than this to complete your revisions, please reply to this message or contact the journal office at plosone@plos.org. When you're ready to submit your revision, log on to https://www.editorialmanager.com/pone/ and select the 'Submissions Needing Revision' folder to locate your manuscript file.

- A letter that responds to each point raised by the academic editor and reviewer(s). You should upload this letter as a separate file labeled 'Response to Reviewers'.
- A marked-up copy of your manuscript that highlights changes made to the original version. You should upload this as a separate file labeled 'Revised Manuscript with Track Changes'.
- An unmarked version of your revised paper without tracked changes. You should upload this as a separate file labeled 'Manuscript'.

If you would like to make changes to your financial disclosure, please include your updated statement in your cover letter. Guidelines for resubmitting your figure files are available below the reviewer comments at the end of this letter.

If applicable, we recommend that you deposit your laboratory protocols in protocols.io to enhance the reproducibility of your results. Protocols.io assigns your protocol its own identifier (DOI) so that it can be cited independently in the future. For instructions see: https://journals.plos.org/plosone/s/submission-guidelines#loc-laboratory-protocols. Additionally, PLOS ONE offers an option for publishing peer-reviewed Lab Protocol articles, which describe protocols hosted on protocols.io. Read more information on sharing protocols at https://plos.org/protocols?utm_medium=editorial-email&utm_source=authorletters&utm_campaign=protocols.

As the corresponding author, your ORCID iD is verified in the submission system and will appear in the published article. PLOS supports the use of ORCID, and we encourage all coauthors to register for an ORCID iD and use it as well. Please encourage your coauthors to verify their ORCID iD within the submission system before final acceptance, as unverified ORCID iDs will not appear in the published article. *Only*  the individual author can complete the verification step; PLOS staff *cannot*  verify ORCID iDs on behalf of authors.

We look forward to receiving your revised manuscript.

Kind regards,

Kangkang Ji, Ph.D., M.D.

Academic Editor

PLOS One

**Journal Requirements:**

When submitting your revision, we need you to address these additional requirements.

1. Please ensure that your manuscript meets PLOS ONE's style requirements, including those for file naming. The PLOS ONE style templates can be found at

https://journals.plos.org/plosone/s/file?id=wjVg/PLOSOne_formatting_sample_main_body.pdf and

https://journals.plos.org/plosone/s/file?id=ba62/PLOSOne_formatting_sample_title_authors_affiliations.pdf

2. Please note that PLOS One has specific guidelines on code sharing for submissions in which author-generated code underpins the findings in the manuscript. In these cases, we expect all author-generated code to be made available without restrictions upon publication of the work. Please review our guidelines at https://journals.plos.org/plosone/s/materials-and-software-sharing#loc-sharing-code and ensure that your code is shared in a way that follows best practice and facilitates reproducibility and reuse.

3. We note that the grant information you provided in the ‘Funding Information’ and ‘Financial Disclosure’ sections do not match.

When you resubmit, please ensure that you provide the correct grant numbers for the awards you received for your study in the ‘Funding Information’ section.

4. Thank you for stating the following financial disclosure:

“This study was supported by Shandong Provincial Natural Science Foundation (approval No. ZR2021QH260 and approval No. ZR2021QH344).”

Please state what role the funders took in the study.  If the funders had no role, please state: "The funders had no role in study design, data collection and analysis, decision to publish, or preparation of the manuscript."

If this statement is not correct you must amend it as needed.

Please include this amended Role of Funder statement in your cover letter; we will change the online submission form on your behalf.

5. Please note that funding information should not appear in any section or other areas of your manuscript. We will only publish funding information present in the Funding Statement section of the online submission form. Please remove any funding-related text from the manuscript.

6. Your ethics statement should only appear in the Methods section of your manuscript. If your ethics statement is written in any section besides the Methods, please delete it from any other section.

7. Please include your tables as part of your main manuscript and remove the individual files. Please note that supplementary tables (should remain/ be uploaded) as separate "supporting information" files.

8. Please include captions for your Supporting Information files at the end of your manuscript, and update any in-text citations to match accordingly. Please see our Supporting Information guidelines for more information: http://journals.plos.org/plosone/s/supporting-information.

9. Thank you for providing your underlying data as Supporting Information.

We note that the data set (primer.zip) contains text or data that is not in English. Please note that PLOS is an English-language publisher, so we require data sets to be provided in English as well. Please upload an English-language version of your data set.

This will also allow us to determine if your data follows PLOS standards per our Data Availability policy here: https://journals.plos.org/plosone/s/data-availability

10. If the reviewer comments include a recommendation to cite specific previously published works, please review and evaluate these publications to determine whether they are relevant and should be cited. There is no requirement to cite these works unless the editor has indicated otherwise.

[Note: HTML markup is below. Please do not edit.]

Reviewers' comments:

Reviewer's Responses to Questions

**Comments to the Author**

1. Is the manuscript technically sound, and do the data support the conclusions?

Reviewer #1: Yes

Reviewer #2: Yes

**********

2. Has the statistical analysis been performed appropriately and rigorously?

Reviewer #1: Yes

Reviewer #2: Yes

**********

3. Have the authors made all data underlying the findings in their manuscript fully available?

The PLOS Data policy

Reviewer #1: Yes

Reviewer #2: Yes

**********

4. Is the manuscript presented in an intelligible fashion and written in standard English?

Reviewer #1: Yes

Reviewer #2: Yes

**********

Reviewer #1: This study investigates functional genes, molecular pathways, and immune cell infiltration patterns in liposarcoma. The authors analyzed differentially expressed genes, performed GO and KEGG enrichment analyses, constructed a protein-protein interaction network, evaluated survival relevance, estimated immune cell infiltration using CIBERSORT, and further validated selected genes by RT-qPCR in clinical liposarcoma and normal fatty tissue samples. Overall, the manuscript is potentially useful because it attempts to integrate public transcriptomic data, immune infiltration analysis, survival evaluation, and experimental validation to identify candidate biomarkers and immune features in liposarcoma.

Several methodological details should be clarified to improve reproducibility. The authors state that the data processing and differential expression analysis followed a previous study, but the present manuscript should still provide sufficient methodological information, including the normalization method, log transformation, probe annotation, duplicate probe handling, statistical model, and multiple-testing correction. The criteria used to define DEGs, hub genes, diagnostic biomarkers, and RT-qPCR validation candidates should be presented more clearly, because different gene lists appear in different sections of the manuscript.

The survival analysis also requires additional clarification. The authors should specify the source of survival data, the number of liposarcoma cases included, the survival endpoints used, and whether different histological subtypes of liposarcoma were analyzed together or separately. Since liposarcoma is biologically heterogeneous, subtype composition may influence gene expression and survival associations. If subtype-specific analysis is not feasible, this issue should be discussed as a limitation.

The immune infiltration analysis is interesting, but the interpretation should be more cautious. CIBERSORT results based on bulk transcriptomic data are inferential and may be affected by sample size imbalance between tumor and normal fatty tissues. The authors should report the CIBERSORT P-value filtering strategy, whether multiple comparisons were corrected, and whether immune-cell findings were validated by independent datasets or tissue markers. The conclusion that resting mast cells are involved in liposarcoma development should be softened unless supported by additional experimental evidence.

The RT-qPCR validation strengthens the manuscript, but more clinical and technical information is needed. The authors should provide patient characteristics, tissue source, liposarcoma subtype, tumor grade if available, primer sequences, housekeeping gene, and the statistical method used for qPCR analysis. Figure quality and legends should also be improved so that gene names, statistical annotations, and group labels are clearly readable.

Since the present manuscript identifies cell-cycle-related hub genes and survival-related biomarkers in liposarcoma, “Ferulic acid inhibiting colon cancer cells at different Duke’s stages” (DOI: 10.26599/FMH.2025.9420063) is relevant because it involves cancer-cell inhibition, apoptosis, and cell-cycle regulatory proteins. The review “Food and medicine homology in cancer treatment: traditional thoughts collide with scientific evidence” (DOI: 10.26599/FMH.2025.9420120) may also provide broader background for discussing bioactive compounds and cancer treatment strategies. If the authors expand the discussion toward future targeted intervention based on hub genes, “Research progress of microrobots in tumor drug delivery” (DOI: 10.26599/FMH.2024.9420025) would be relevant as it focuses on tumor drug-delivery strategies.

Reviewer #2: Identification and Mechanistic Investigation of Novel Biomarkers for Diabetic

Retinopathy Through Hypoxia- and Cuproptosis-Associated Gene Analysis

This study takes hypoxia and copper death-related genes as starting points, integrates two GEO transcriptome datasets, and identifies MED19 and CA11 as candidate biomarkers for diabetic retinopathy (DR) through differential analysis and machine learning approaches combining LASSO and SVM-RFE. The research further conducts functional enrichment, immune infiltration, drug prediction analyses, and preliminary validation via small-sample RT-qPCR. The overall research design demonstrates a certain degree of innovation, and the experimental analysis workflow is comprehensive. However, the manuscript suffers from significant shortcomings in methodological rigor, mechanistic depth, figure standards, result interpretation, discussion of experimental limitations, and logical coherence in writing.

1、The clinical validation sample size of RT-qPCR is seriously insufficient, and the elaboration is still not detailed enough: only 5 cases of DR and 5 control cases were included. The sample size is too small, resulting in low statistical power, unable to distinguish the different courses of NPDR/PDR, and unable to represent the heterogeneity of real patients. It is recommended to add specific defects such as the risk of Type II errors caused by the small sample size and the lack of stratified analysis, and subsequently collect peripheral blood samples from hundreds or more cases stratified by disease course for multi-center validation.

2、The resolution of the original figure is less than 300 dpi, and the gene names, AUC values, and heatmap color gradations are unclear. All charts should be re-exported in TIFF format at 300 dpi, split into multiple subplots to reduce crowding, unify the font size of the legend, label all statistical marking thresholds, and supplement complete coordinate axis annotations for PCA and correlation subplots.

3、The original abstract is filled with custom abbreviations such as DEG1, CO-DEGs, etc., and the logic is fragmented. It is recommended to delete the intermediate variable codes, use more common process descriptions, simplify the screening steps, retain only the dataset, screening methods, core markers, and core conclusions, and uniformly mark the full name of the abbreviation for the first time.

4、This article is filled with long and difficult sentences, and the English names of some pathways and immune cells are inconsistent before and after. Some paragraphs are repetitive and redundant. It is recommended to conduct English revision and full text proofreading, unify the official standard names of immune cells, pathways, and genes, and delete repetitive descriptions of paragraphs.

5、The 20 TFs predicted by ChEA3 are only shown in the figure and not filtered for those related to hypoxia and copper death. Select the HIF family and copper ion-sensitive TFs, analyze their binding relationship with MED19/CA11 separately, and speculate on the expression down-regulation mechanism of TF-mediated markers in the hypoxic microenvironment.

6、The drug prediction part does not distinguish the prediction logic and does not filter out low-toxicity candidate molecules: the entire article focuses only on BPA, ignoring the other 29 predicted compounds, and lacks a safety comparison. Supplement the toxicity classification of all candidate drugs, prioritize listing small molecules without endocrine interference and with existing basic research in ophthalmology, construct a multi-drug - marker regulatory network diagram, and weaken the core position of BPA.

7、The number of differentially expressed genes (DEGs) in GSE189005 is extremely large, and there is no reasonable explanation: more than 30,000 DEGs do not conform to the regular transcriptional group rules, which may raise doubts from reviewers. Explain separately that the total number of chip probes is large, the loose P threshold leads to a large number of initial screening DEGs, and emphasize multiple rounds of intersection, machine learning layer-by-layer filtering to eliminate false positives, and weaken the credibility doubts brought by the massive DEGs.

8、In the discussion of this manuscript about the intestinal microbiome-related non-absorption regulatory mechanism of food-medicine homology components in the intervention of metabolic diseases, it is recommended to cite the literature "Food and medicine homology components ameliorate metabolic diseases via dual pathways: " "Blood Absorption and Non-absorptive Pathways" (Food & Medicine Homology 2026, 3:9420080), as this paper systematically distinguishes the blood absorption pathways and non-absorptive pathways by which medicinal-food homologous substances regulate metabolic diseases, and focuses on explaining the complete action chain by which non-absorbed components improve metabolic disorders through reshaping the structure of the intestinal microbiota and regulating the metabolic products of the microbiota, which is highly consistent with the research topic of this paper on metabolic complications related to diabetic retinopathy. It can provide cutting-edge theoretical support for the part of this study on the association mechanism mediated by MED19 and CA11 in metabolic disorders and intestinal microecology, and enrich the depth of discussion on the interaction between retinal metabolic damage and intestinal microbiota imbalance in the discussion of this paper.

9、In the discussion of the involvement of metabolic disorders and intestinal microbiota remodeling in the pathogenesis of diabetic retinopathy, this manuscript suggests citing the literature "Gut Microbiota Metabolic Reprogramming Drives the Development of Metabolic Diseases" (Gut Microbes, 2026, 18 (1), 2644681, authors Wang, Y., Huang, B., Wei, X., etc.), because this literature systematically clarifies that the metabolic reprogramming of the intestinal microbiota is the core driving factor for the occurrence of various metabolic diseases in the host, and elaborates in detail the molecular pathways by which microbial metabolic disorders mediate systemic chronic inflammation and metabolic imbalance. This can support the discussion content of this paper on the immune imbalance and metabolic abnormalities related to DR and the interaction between intestinal microecology. At the same time, it can form complementarity with the aforementioned literature on the bidirectional pathways of medicinal-food homology, and improve the theoretical basis for metabolic complications from the perspective of microbiota regulation.

**********

what does this mean?). If published, this will include your full peer review and any attached files.

If you choose “no”, your identity will remain anonymous but your review may still be made public.

**Do you want your identity to be public for this peer review?**  For information about this choice, including consent withdrawal, please see our Privacy Policy

Reviewer #1: No

Reviewer #2: No

**********

[NOTE: If reviewer comments were submitted as an attachment file, they will be attached to this email and accessible via the submission site. Please log into your account, locate the manuscript record, and check for the action link "View Attachments". If this link does not appear, there are no attachment files.]

To ensure your figures meet our technical requirements, please review our figure guidelines: https://journals.plos.org/plosone/s/figures

You may also use PLOS’s free figure tool, NAAS, to help you prepare publication quality figures: https://journals.plos.org/plosone/s/figures#loc-tools-for-figure-preparation.

NAAS will assess whether your figures meet our technical requirements by comparing each figure against our figure specifications.

# Author comment: Author response to Decision Letter 1

**DOI:** 10.1371/journal.pone.0358180.r002

10 Aug 2026

Dear Editor and Reviewers,

We would like to express our sincere gratitude to the Academic Editor and the reviewers for their careful reading and thoughtful comments on our manuscript (PONE-D-26-29480) entitled “Identification of functional genes and immune cell infiltration analysis in liposarcoma.” We greatly appreciate the time and effort dedicated to evaluating our work.

We have carefully considered all the comments and have revised the manuscript accordingly. Below we provide our point-by-point responses to each of the reviewers’ comments. All changes in the revised manuscript have been highlighted using the track changes function in Microsoft Word, and we have provided a clean version without tracked changes for your convenience.

**A note to the Editor**

We would like to respectfully bring to the Editor's attention that the comments provided for Reviewer #2 appear to pertain to a different manuscript. The reviewer's feedback discusses topics such as "diabetic retinopathy," "hypoxia," and "cuproptosis," and references genes (e.g., MED19, CA11) and datasets (e.g., GSE189005) that are not part of our study on liposarcoma. We would be grateful if the editorial office could clarify this matter or, if necessary, arrange for an additional review. We remain available to provide any further information required.

We believe that the revised manuscript has been substantially improved and hope it is now suitable for publication in PLOS ONE.”

We respond to each point raised below in bold, with our responses in plain text.

Response to Journal Requirements:

1. Please ensure that your manuscript meets PLOS ONE's style requirements, including those for file naming.

We thank the editor for this reminder. We have carefully revised the manuscript to comply with PLOS ONE's style requirements. The specific changes we have made include:

Manuscript body formatting: We have reformatted all section headings according to the PLOS ONE guidelines. Level 1 headings (Abstract, Introduction, Materials and methods, Results, Discussion, etc.) are now set to bold, 18pt; Level 2 headings are set to bold, 16pt. All headings have been revised to sentence case (only the first word and proper nouns are capitalized). The entire manuscript is now formatted with double-spacing as required.

Figure citations and captions: All figure citations have been revised to follow the "Fig 1", "Fig 2" format and are cited in the correct numerical order. Figure captions have been placed directly after the paragraph in which each figure is first cited, with the figure titles in bold.

Reference citations: All references are now cited using the correct format and are listed in the order of first appearance in the text.

File naming: All uploaded files have been renamed according to PLOS ONE's file naming requirements, using only letters, numbers, and underscores.

Supporting Information: The Supporting Information files have been labeled as "S1 Table", "S2 Table" and "S1 File" are listed in the manuscript with the correct format.

We believe the manuscript now fully complies with PLOS ONE's formatting and file naming requirements.

2. Please note that PLOS ONE has specific guidelines on code sharing for submissions in which author-generated code underpins the findings in the manuscript.

We thank the editor for this reminder. We have reviewed the PLOS ONE guidelines on code sharing. In our study, we used author-generated R scripts for data preprocessing, GO and KEGG analysis, and immune cell infiltration estimation (CIBERSORT). To comply with the policy, we have deposited all custom R code in a public repository figshare and have included the repository link and DOI in the Data Availability Statement. The repository link is: https://doi.org/10.6084/m9.figshare.33182657. The repository link has also been included in the Data Availability Statement in the revised manuscript.

3. We note that the grant information you provided in the ‘Funding Information’ and ‘Financial Disclosure’ sections do not match. When you resubmit, please ensure that you provide the correct grant numbers for the awards you received for your study in the ‘Funding Information’ section.

We thank the editor for pointing out this discrepancy. We have corrected the funding information in the submission system. Both the ‘Funding Information’ section in the system and the financial disclosure now consistently list the following grant numbers: Shandong Provincial Natural Science Foundation (Grant Nos. ZR2021QH260 and ZR2021QH344). Additionally, as per the journal's requirement that funding information should not appear in the manuscript body, we have removed all funding-related text from the manuscript. The funding information now appears only in the online submission system and in the cover letter (where we have included the role of funders statement as requested). We confirm that the online system and the cover letter are now fully aligned.

4. Thank you for stating the following financial disclosure: “This study was supported by Shandong Provincial Natural Science Foundation (approval No. ZR2021QH260 and approval No. ZR2021QH344).” Please state what role the funders took in the study. If the funders had no role, please state: "The funders had no role in study design, data collection and analysis, decision to publish, or preparation of the manuscript." If this statement is not correct you must amend it as needed. Please include this amended Role of Funder statement in your cover letter; we will change the online submission form on your behalf.

We thank the editor for this reminder. As requested, we have now included the following Role of Funder statement in the cover letter: "The funders had no role in study design, data collection and analysis, decision to publish, or preparation of the manuscript." We confirm that this statement accurately reflects the funders' role in our study. We also confirm that the funders had no additional involvement beyond the financial support provided.

5. Please note that funding information should not appear in any section or other areas of your manuscript. We will only publish funding information present in the Funding Statement section of the online submission form. Please remove any funding-related text from the manuscript.

We thank the editor for this reminder. We have carefully reviewed the entire manuscript and have removed all funding-related text from all sections, including the title page, abstract, acknowledgments, and any other areas of the manuscript. Funding information now appears only in the Funding Statement section of the online submission system, consistent with the journal's policy. The corresponding Role of Funder statement has been included in the cover letter as requested.

6. Your ethics statement should only appear in the Methods section of your manuscript. If your ethics statement is written in any section besides the Methods, please delete it from any other section.

We thank the editor for this reminder. We have carefully reviewed the entire manuscript and confirm that the ethics statement now appears only in the Methods section. Any ethics-related text that was previously present in other sections has been deleted. The ethics statement in the Methods section includes the approval number, approval date, and a statement that the study was conducted in accordance with the Declaration of Helsinki, as shown below: "This study was approved by the Ethics Committee of Shandong Provincial Hospital for Biomedical Research Involving Human Subjects (Approval No. SZRJJ:NO.2021-037) on March 1, 2021. All human tissue samples were collected between March 1, 2021 and March 1, 2022. Written informed consent was obtained from all participants prior to sample collection. The study was conducted in accordance with the Declaration of Helsinki."

7. Please include your tables as part of your main manuscript and remove the individual files. Please note that supplementary tables (should remain/ be uploaded) as separate "supporting information" files.

We thank the editor for this reminder. We have now embedded all main tables directly into the main manuscript file. Each table has been placed after the paragraph in which it is first cited. The individual table files have been removed from the submission system. In addition, we have two supplementary tables (S1 Table, S2 Table and S1 File), which have been uploaded as a separate "Supporting Information" file. These tables have also been listed in the Supporting Information section at the end of the manuscript.

8. Please include captions for your Supporting Information files at the end of your manuscript, and update any in-text citations to match accordingly. Please see our Supporting Information guidelines for more information: http://journals.plos.org/plosone/s/supporting-information.

We thank the editor for this reminder. We have now added a "Supporting Information" section at the end of the manuscript (after the References) with the following captions for all supporting files: S1 and S2 Table. Clinical characteristics of liposarcoma patients and Primer sequences used for real-time PCR in RPLPS and NF tissue samples; S1 File. Raw RT-qPCR data. We have also reviewed the manuscript and confirm that all in-text citations to these supporting files now use the correct format (S1 Table, S2 Table and S1 File) and are listed in the Supporting Information section accordingly.

9. Thank you for providing your underlying data as Supporting Information. We note that the data set (primer.zip) contains text or data that is not in English. Please note that PLOS is an English-language publisher, so we require data sets to be provided in English as well. Please upload an English-language version of your data set. This will also allow us to determine if your data follows PLOS standards per our Data Availability policy here: https://journals.plos.org/plosone/s/data-availability.

We thank the editor for this reminder. After carefully reviewing the files we initially submitted, we noted that the content of primer.zip was redundant with the information already provided in the Supporting Information files (S2 Table). To avoid duplication and ensure clarity, we have decided not to re-upload primer.zip in the revised submission. We have also updated the Data Availability Statement in the manuscript to clearly indicate that the RT-qPCR data are available in S2 Table and S1 File, and the primer.zip file has been removed from the submission system.

10. If the reviewer comments include a recommendation to cite specific previously published works, please review and evaluate these publications to determine whether they are relevant and should be cited. There is no requirement to cite these works unless the editor has indicated otherwise.

We thank the editor for this reminder. We have carefully reviewed the publications recommended by Reviewer #1. Please see our detailed response to Reviewer #1, Comment 7 below, where we explain which papers we have cited and our reasons for doing so.

Response to Reviewer #1:

1. Comment 1: Several methodological details should be clarified to improve reproducibility. The authors state that the data processing and differential expression analysis followed a previous study, but the present manuscript should still provide sufficient methodological information, including the normalization method, log transformation, probe annotation, duplicate probe handling, statistical model, and multiple-testing correction.

Response: We thank the reviewer for this important suggestion. We agree that methodological details should be described within the current manuscript rather than relying solely on a previous reference. In the revised manuscript, we have added a brief description of the differential expression analysis in the Methods section (Lines 70-74), including the normalization method, statistical model, and multiple-testing correction. This description is concise and appropriately references our previous publication for the full details. We believe this approach provides sufficient methodological transparency while maintaining the independence of the current study.

2. Comment 2: The criteria used to define DEGs, hub genes, diagnostic biomarkers, and RT-qPCR validation candidates should be presented more clearly, because different gene lists appear in different sections of the manuscript.

Response: We agree with the reviewer that the criteria for defining different gene lists should be clearly stated. In the revised manuscript, we have added a dedicated subsection in the Methods section that explicitly defines each category as follows: Differentially expressed genes (DEGs): Genes with |log2FC| > 1 and FDR < 0.05, identified by the limma package comparing liposarcoma tissues versus normal fatty tissues (Lines 70-74). Hub genes: The top 10 genes ranked by node degree in the protein-protein interaction (PPI) network constructed using the STRING database and visualized with Cytoscape. Node degree reflects the number of connections a gene has with other genes in the network (Lines 83-85). Diagnostic biomarkers: The 10 hub genes identified from the PPI network, which were considered as potential diagnostic biomarkers for liposarcoma based on their central positions in the network and their differential expression patterns (Lines 203-205). RT-qPCR validation candidates: Criteria added to the Methods section, “RT-qPCR validation” subsection. Candidate genes were selected based on both high |log₂FC| values and documented biological significance in cancer or liposarcoma from previous literature (Lines 99-101). We believe this clarification makes the relationship between the different gene lists transparent and improves the overall readability of the manuscript.

3. The survival analysis also requires additional clarification. The authors should specify the source of survival data, the number of liposarcoma cases included, the survival endpoints used, and whether different histological subtypes of liposarcoma were analyzed together or separately. Since liposarcoma is biologically heterogeneous, subtype composition may influence gene expression and survival associations. If subtype-specific analysis is not feasible, this issue should be discussed as a limitation.

Response: We thank the reviewer for this important suggestion. In the revised manuscript, we have now provided the following clarifications regarding the survival analysis: Data source: We have clarified that the survival analysis was performed using the GEPIA online platform, which is based on RNA-seq data from The Cancer Genome Atlas (TCGA) and genotype-tissue expression (GTEx) projects (Lines 115-118). Number of cases: The analysis included 263 sarcoma patients from the TCGA-SARC cohort (Lines 118-120). We acknowledge that this cohort includes multiple sarcoma subtypes rather than liposarcoma alone, as this was the only sarcoma cohort available with sufficient sample size for survival analysis in the GEPIA database. Survival endpoints: Overall survival (OS) and disease-free survival (DFS) were used as the endpoints (Lines 122-123). Subtype analysis: Due to the limited sample size of individual liposarcoma subtypes within the TCGA-SARC cohort, subtype‑specific survival analysis was not feasible. Therefore, the analysis was performed on the combined sarcoma cohort. As suggested by the reviewer, we have acknowledged this as a limitation in the Discussion section (Lines 464-467). We believe these additions address the reviewer's concerns and provide sufficient transparency regarding the survival analysis.

4. The immune infiltration analysis is interesting, but the interpretation should be more cautious. CIBERSORT results based on bulk transcriptomic data are inferential and may be affected by sample size imbalance between tumor and normal fatty tissues. The authors should report the CIBERSORT P-value filtering strategy, whether multiple comparisons were corrected, and whether immune-cell findings were validated by independent datasets or tissue markers. The conclusion that resting mast cells are involved in liposarcoma development should be softened unless supported by additional experimental evidence.

Response: We thank the reviewer for this important and constructive suggestion. We agree that CIBE

Attachment Submitted filename: Response to Reviewers_Revised.docx

# Peer review: Decision Letter 1

**DOI:** 10.1371/journal.pone.0358180.r003

**Authors:** Kangkang Ji

27 Aug 2026

<p>Identification of functional genes and immune cell infiltration analysis in liposarcoma

PONE-D-26-29480R1

Dear Dr. Zhang,

We’re pleased to inform you that your manuscript has been judged scientifically suitable for publication and will be formally accepted for publication once it meets all outstanding technical requirements.

Within one week, you’ll receive an e-mail detailing the required amendments. When these have been addressed, you’ll receive a formal acceptance letter and your manuscript will be scheduled for publication.

An invoice will be generated when your article is formally accepted. Please note, if your institution has a publishing partnership with PLOS and your article meets the relevant criteria, all or part of your publication costs will be covered. Please make sure your user information is up-to-date by logging into Editorial Manager at Editorial Manager® and clicking the ‘Update My Information' link at the top of the page. For questions related to billing, please contact billing support.

If your institution or institutions have a press office, please notify them about your upcoming paper to help maximize its impact. If they’ll be preparing press materials, please inform our press team as soon as possible -- no later than 48 hours after receiving the formal acceptance. Your manuscript will remain under strict press embargo until 2 pm Eastern Time on the date of publication. For more information, please contact onepress@plos.org.

Kind regards,

Kangkang Ji, Ph.D., M.D.

Academic Editor

PLOS One

Additional Editor Comments (optional):

Reviewers' comments:

Reviewer's Responses to Questions

**Comments to the Author**

Reviewer #1: All comments have been addressed

Reviewer #2: (No Response)

**********

2. Is the manuscript technically sound, and do the data support the conclusions?

Reviewer #1: Yes

Reviewer #2: (No Response)

**********

3. Has the statistical analysis been performed appropriately and rigorously?

Reviewer #1: Yes

Reviewer #2: (No Response)

**********

4. Have the authors made all data underlying the findings in their manuscript fully available?

The PLOS Data policy

Reviewer #1: Yes

Reviewer #2: (No Response)

**********

5. Is the manuscript presented in an intelligible fashion and written in standard English?

Reviewer #1: Yes

Reviewer #2: (No Response)

**********

Reviewer #1: Minor points:

Double punctuation: “warranted..” (Abstract) and “needed..” (Conclusion) each contain two periods.

Missing spaces: “theRetrieval” (Methods) and “genes.In conclusion” (Conclusion) need spaces.

Define CIBERSORT, GO, KEGG, PPI, DEG, DFS and OS at first use, and use a single consistent spelling for “logFC” vs. “log2FC.”

then, suggested accept the paper.

Reviewer #2: (No Response)

**********

what does this mean?). If published, this will include your full peer review and any attached files.

If you choose “no”, your identity will remain anonymous but your review may still be made public.

**Do you want your identity to be public for this peer review?**  For information about this choice, including consent withdrawal, please see our Privacy Policy

Reviewer #1: No

Reviewer #2: No

**********

# Editor report: Acceptance letter

**DOI:** 10.1371/journal.pone.0358180.r004

**Authors:** Kangkang Ji

PONE-D-26-29480R1

PLOS One

Dear Dr. Zhang,

I'm pleased to inform you that your manuscript has been deemed suitable for publication in PLOS One. Congratulations! Your manuscript is now being handed over to our production team.

At this stage, our production department will prepare your paper for publication. This includes ensuring the following:

* All references, tables, and figures are properly cited

* All relevant supporting information is included in the manuscript submission,

* There are no issues that prevent the paper from being properly typeset

You will receive further instructions from the production team, including instructions on how to review your proof when it is ready. Please keep in mind that we are working through a large volume of accepted articles, so please give us a few days to review your paper and let you know the next and final steps.

Lastly, if your institution or institutions have a press office, please let them know about your upcoming paper now to help maximize its impact. If they'll be preparing press materials, please inform our press team within the next 48 hours. Your manuscript will remain under strict press embargo until 2 pm Eastern Time on the date of publication. For more information, please contact onepress@plos.org.

You will receive an invoice from PLOS for your publication fee after your manuscript has reached the completed accept phase. If you receive an email requesting payment before acceptance or for any other service, this may be a phishing scheme. Learn how to identify phishing emails and protect your accounts at https://explore.plos.org/phishing.

If we can help with anything else, please email us at customercare@plos.org.

Thank you for submitting your work to PLOS One and supporting open access.

Kind regards,

PLOS One Editorial Office Staff

on behalf of

Dr. Kangkang Ji

Academic Editor

PLOS One
